# Fractional Deletion of Compound Kushen Injection Indicates Cytokine Signaling Pathways are Critical for its Perturbation of the Cell Cycle

**DOI:** 10.1038/s41598-019-50271-4

**Published:** 2019-10-02

**Authors:** T. N. Aung, S. Nourmohammadi, Z. Qu, Y. Harata-Lee, J. Cui, H. Y. Shen, A. J. Yool, T. Pukala, Hong Du, R. D. Kortschak, W. Wei, D. L. Adelson

**Affiliations:** 1 Department of Molecular and Biomedical Science, School of Biological Sciences, University of Adelaide, Adelaide, South Australia 5005 Australia; 2 Adelaide Medical School, University of Adelaide, Adelaide, South Australia 5005 Australia; 3 School of Physical Sciences, University of Adelaide, Adelaide, South Australia 5005 Australia; 4 School of Chinese Materia Medica, Beijing University of Chinese Medicine, Beijing, 100029 P.R. China; 5 Beijing Zhendong Guangming Pharmaceutical Research Institute, Shanxi – Zhendong Pharmaceutical Co Ltd, Beijing, P.R. China

**Keywords:** Drug development, Cellular signalling networks

## Abstract

We used computational and experimental biology approaches to identify candidate mechanisms of action of aTraditional Chinese Medicine, Compound Kushen Injection (CKI), in a breast cancer cell line (MDA-MB-231). Because CKI is a complex mixture of plant secondary metabolites, we used a high-performance liquid chromatography (HPLC) fractionation and reconstitution approach to define chemical fractions required for CKI to induce apoptosis. The initial fractionation separated major from minor compounds, and it showed that major compounds accounted for little of the activity of CKI. Furthermore, removal of no single major compound altered the effect of CKI on cell viability and apoptosis. However, simultaneous removal of two major compounds identified oxymatrine and oxysophocarpine as critical with respect to CKI activity. Transcriptome analysis was used to correlate compound removal with gene expression and phenotype data. Many compounds in CKI are required to trigger apoptosis but significant modulation of its activity is conferred by a small number of compounds. In conclusion, CKI may be typical of many plant based extracts that contain many compounds in that no single compound is responsible for all of the bioactivity of the mixture and that many compounds interact in a complex fashion to influence a network containing many targets.

## Introduction

Natural compounds are chemically diverse and have long served as resources for the identification of drugs^1^. However, the standard approach of fractionating natural product extracts to identify a single compound’s biological activity can fail because the original activity of the mixture is not present in single compounds after fractionation. This failure to identify single compounds implies that some natural product mixtures derive their activity from the interaction of several bioactive compounds within the mixture. Characterising the mode of action of natural product mixtures has remained a difficult task as the combinatorial complexity of such mixtures makes it unfeasible to screen all combinations of the compounds in the mixture.

We introduce here a “subtractive fractionation approach” using high performance liquid chromatography (HPLC) that can pinpoint significant interacting compounds within a mixture when coupled with a suitable bioassay. We combined this approach with RNA sequencing (RNAseq) characterisation of our bioassay, correlating the removal of interacting compounds with concomitant alterations in gene expression. This combination allowed us to identify specific combinations of compounds associated with specific pathways and regulatory interactions. In this report, we have applied this approach for the first time to a particular Traditional Chinese Medicine formulation: CKI, which is used to treat approximately 30,000 cancer patients/day in China in conjunction with Western chemotherapy.

CKI is composed primarily of alkaloids and flavonoids extracted from two herbal medicinal plants: Kushen (*Sophora flavescens*) and Baituling (*Heterosmilax chinensis*). Twenty-one chromatographic peaks have been identified from CKI with eight compounds being recognised as major components on the basis of their abundance^2^. The extract containing the most abundant compounds in CKI is derived from Kushen herb which has a long history in the treatment of patients suffering immune function disorders^3,4^. The main component of CKI, macrozamin, is a derivative of baituling which has been a suggested therapeutic agent for the treatment of inflammatory disease^5^. Gao and colleagues showed that treatment with each of four of the main compounds of CKI (oxymatrine, matrine, sophoridine and N-methylcytisine) at 4 mg/ml significantly decreased cell viability^6^. However, these concentrations are relatively high when compared to the contributing concentration of these four main compounds in CKI^2^. The two main components of CKI, matrine and oxymatrine, may have significant anti-cancer activities in various types of solid tumors including breast cancer, non-small cell lung cancer, cervical cancer, prostate cancer, synovial sarcoma, and hepatocellular carcinoma^7–13^. In contrast, the toxicity of medicinal herbs containing matrine and oxymatrine as main components has also been reported^14^. Administration of matrine 150 mg/kg and oxymatrine 360 mg/kg significantly increased cytochrome P450 family protein CYPB1/2 in rats demonstrating a potential therapeutic drawback of these two compounds^15^. Overall, understanding the effects of CKI based on the effects of single compounds present in CKI has been at best partially successful.

Alternatively, by removing one, two or three compounds, we have been able to map the effects of these compounds and their interactions to effects on specific pathways based on altered gene expression profiles in a cell-based assay. This has illuminated the roles of several major compounds of CKI, which on their own have little or no activity in our bioassay. This approach can be used to dissect the roles and interactions of individual compounds from complex natural compound mixtures whose biological activity cannot be attributed to single purified compounds.

## Results

### Subtractive fractionation overview

**Figure 1:**
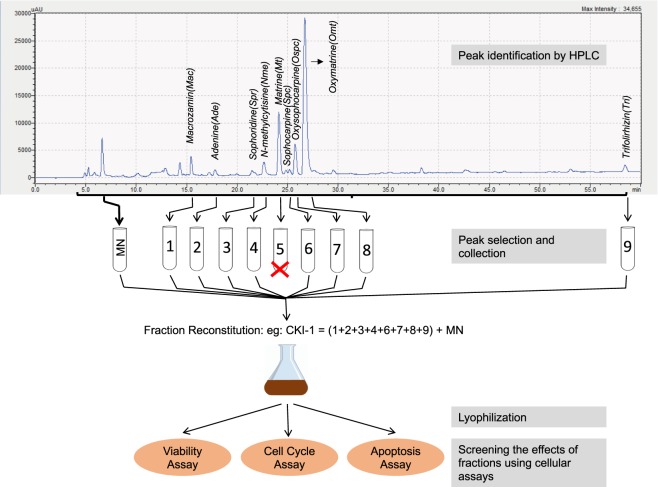
Fractionation of CKI. Diagram illustrating the process of subtractive fractionation, reconstitution, and screening of fractionated compounds using three cell-based assays.

Well-resolved chromatographic separation of CKI was used to collect all of the major components of CKI as individual fractions (Fig. 1). We then reconstituted all of the separated fractions except for those we wished to subtract. We tested the reconstituted combination of compounds/peaks to see if removal of a single (CKI-1) or multiple compounds, (CKI-2 or CKI-3), or removal of all major peaks (minor, MN) or depletion of all minor peaks (major, MJ) significantly altered the effect of CKI in our cell based assays. Our cell based assays^16^ measured MDA-MB-231 (human breast adenocarcinoma) cell viability, cell-cycle phase and cell apoptosis. A summary of the subtractive fractions used in the cell-based assays is shown in Table 1. We then carried out RNA isolation of cells treated with CKI, individual compounds or CKI deletions for RNAseq. Differentially expressed (DE) genes in these samples allowed the association of specific compounds with cell phenotype and underlying alterations in gene regulation. By comparing DE genes across treatment combinations, we identified specific candidate pathways that were altered by removal of single or multiple compounds, as detailed below.

**Table 1:** Summarised results of HPLC fractionation and treatments using three cell-based assays at 48-hour from Figs 2 and 3 and Supplementary Fig. 2.

HPLC Fractionation	Treatments	Proliferation Assay (MDA-MB-213)	Cell-Cycle Assay (MDA-MB-213)	Apoptosis Assay in Three Cell Lines		
				MDA-MB-231	HEK-293	HFF
9 known + small unknown	Original CKI	***	****	**	**	
9 known major compounds	MJ					
Small unknown minor compounds	MN	***				
CKI-1	CKI-Mac					
	CKI-Ade					
	CKI-Tri					
	CKI-Nme					
	CKI-Spr					
	CKI-Mt					
	CKI-Omt					
	CKI-Ospc					
	CKI-Spc					
CKI-3	CKI-MacAdeTri					
	CKI-MtNmeSpr					
	CKI-OmtOspcSpc					
	CKI-MacOmtOspc	*	****	****	****	*
CKI-2	CKI-MacAde					
	CKI-MacTri					
	CKI-AdeTri					
	CKI-MtNme					
	CKI-MtSpr					
	CKI-NmeSpr					
	CKI-OmtOspc	*	****	****	****	**
	CKI-OmtSpc					
	CKI-OspcSpc					
3 compounds	MacAdeTri					
	MtNmeSpr					
	OmtOspcSpc					
	MacOmtOspc					
2 compounds	MacAde					
	MacTri					
	AdeTri					
	MtNme					
	MtSpr					
	NmeSpr					
	OmtOspc					
	OmtSpc					
	OspcSpc					

Statistically significant results of CKI treatment were calculated based on comparison against UT whereas those of other treatments were calculated based on comparison against corresponding CKI treatments. Statistically significant results were represented as (*)P < 0.05 or (**)P < 0.01 or (***)P < 0.001 or (****)P < 0.0001.

### HPLC fractions and content identification using LC-MS/MS

HPLC fractionation and reconstitution was used to generate a number of CKI-1, CKI-2, CKI-3, MJ and MN mixtures, (Fig. 1 and Supplementary Fig. 1) with specific combinations and their components shown in Table 1. The concentrations of known compounds in CKI and reconstituted subtractive fractions were determined from standard curves (Supplementary Data 1) for nine available reference compounds, using cytisine as an internal standard (Table 2). The combined concentration of nine reference compounds from CKI was approximately 10.8 mg/ml, whereas subtractive fractions CKI-OmtOspc and CKI-MacOmtOspc had concentrations of reference compounds of 3.8 mg/ml and 2.1 mg/ml which were equivalent to the concentrations of these compounds in unfractionated CKI. The depleted OmtOspc and MacOmtOspc were not observed in the CKI-OmtOspc and CKI-MacOmtOspc respectively. Based on the concentrations of nine measurable major compounds from CKI, CKI-OmtOspc and CKI-MacOmtOspc (Table 2), the observed final concentration of nine reference compounds in 1/13.25 dilution of CKI, CKI-OmtOspc and CKI-MacOmtOspc that cells were exposed to was 0.82 mg/ml, 0.29 mg/ml and 0.16 mg/ml respectively. These collectively suggested any effects observed after the treatments of CKI-OmtOspc and CKI-MacOmtOspc were not influenced by the concentrations. A total of 9 (CKI-1), 4 (CKI-3) and 9 (CKI-2) combinations, along with MJ and MN deletions were tested in our cell-based assays (Table 1).

**Table 2:** Concentration of nine major compounds in CKI (Batch No: 20170322), remaining major compounds in CKI-OmtOspc, and remaining major compounds in CKI-MacOmtOspc.

Mixtures	Compounds	Regression	Coefficient of Determination	Concentration (mg/ml) (n = 2)
CKI	Macrozamin	y = 6E-05x + 6E-05	0.996	1.1 ± 0.03
	Adenine	y = 0.021x + 0.0074	0.994	0.09 ± 0.09
	N-methylcytisine	y = 0.0937x + 0.042	0.9895	0.17 ± 0.02
	Sophoridine	y = 0.1443x + 0.2679	0.987	0.4 ± 0.08
	Matrine	y = 0.0132x + 0.7512	0.993	1.26 ± 0.06
	Sophocarpine	y = 0.0343x + 0.3974	0.994	0.54 ± 0.02
	Oxysophocarpine	y = 0.0371x − 0.0108	0.999	1.1 ± 0.1
	Oxymatrine	y = 0.0132x + 0.6226	0.992	6.1 ± 0.09
	Trifolirhizin	y = 0.0026x − 0.0002	0.990	0.08 ± 0.002
	**Total**			**10.8**
CKI-OmtOspc	Macrozamin	y = 6E-05x + 6E-05	0.996	1.1 ± 0.04
	Adenine	y = 0.021x + 0.0074	0.994	0.3 ± 0.5
	N-methylcytisine	y = 0.0937x + 0.042	0.9895	0.03 ± 0.03
	Sophoridine	y = 0.1443x + 0.2679	0.987	0.3 ± 0.07
	Matrine	y = 0.0132x + 0.7512	0.993	1.7 ± 0.1
	Sophocarpine	y = 0.0343x + 0.3974	0.994	0.3 ± 0.01
	Trifolirhizin	y = 0.0026x–0.0002	0.990	0.07 ± 0.01
	**Total**			**3.8**
CKI-MacOmtOspc	Adenine	y = 0.021x + 0.0074	0.994	0.06 ± 0.09
	N-methylcytisine	y = 0.0937x + 0.042	0.9895	0.02 ± 0.01
	Sophoridine	y = 0.1443x + 0.2679	0.987	0.04 ± 0.02
	Matrine	y = 0.0132x + 0.7512	0.993	1.7 ± 0.06
	Sophocarpine	y = 0.0343x + 0.3974	0.994	0.2 ± 0.02
	Trifolirhizin	y = 0.0026x–0.0002	0.990	0.08 ± 0.003
	**Total**			**2.1**

*Total alkaloid content in CKI = 26.5 mg/ml based on manufacturer’s assay.

### Phenotypic changes associated with compound deletion

**Figure 2:**
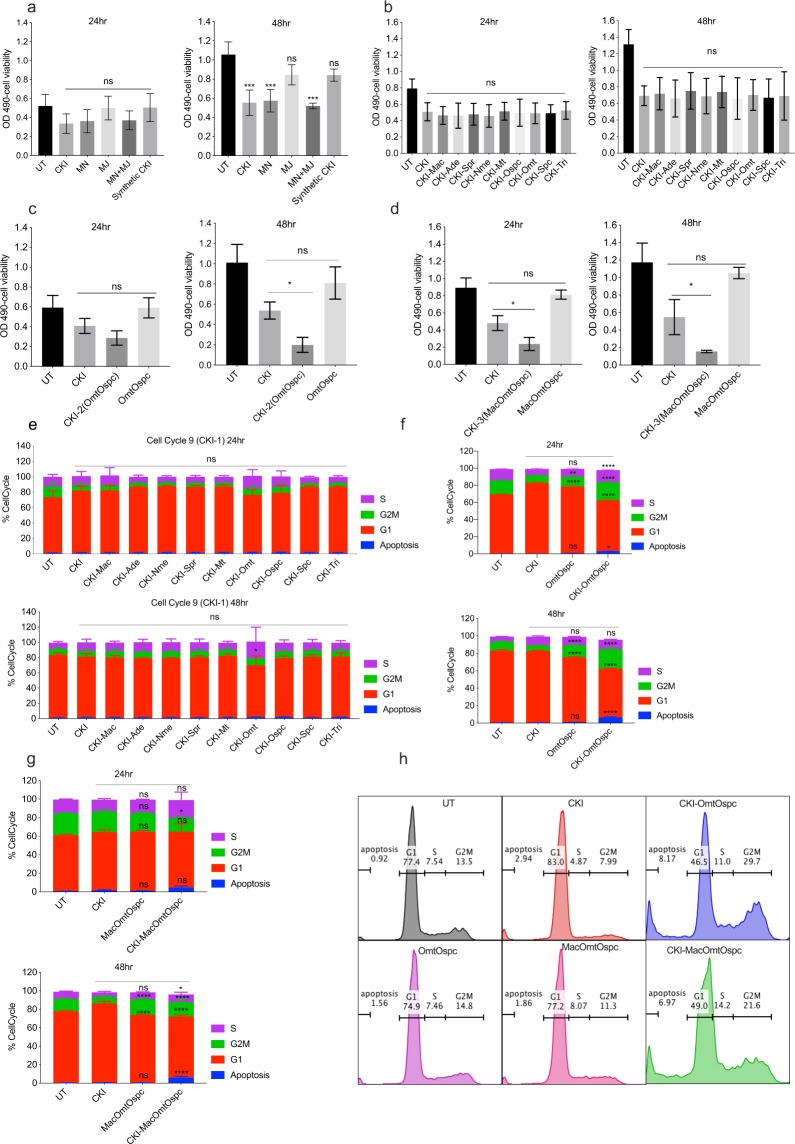
XTT cell viability assay and cell-cycle assay of subtractive fractions at 24- and 48-hour timepoint treatments in MDA-MB-231 cells treated with 2 mg/ml of CKI and 2 mg/ml equivalent concentrations of all other treating agents. (**a**) Suppression of cell viability from the following fractions: UT (untreated), MJ, MN, MJ + MN (combination of MJ and MN) and Syn_CKI (synthetic CKI generated using nine major compounds). (**b**) Effect of 9 (CKI-1) subtractive fractions compared to CKI. (**c**) Effect of CKI-OmtOspc subtractive fraction and OmtOspc compared to CKI. (d) Effect of CKI-MacOmtOspc subtractive fraction and MacOmtOspc, compared to CKI. Statistically significant results relative to CKI treatment shown as (*)P < 0.05 or ns (not significant), all data were shown as mean ± SD. (**e**) Effect of 9 (CKI-1) subtractive fractions on cell-cycle in MDA-MB-231 cells as determined by PI staining assay. (**f**) Effect of CKI-OmtOspc subtractive fraction and OmtOspc on cell-cycle in MDA-MB-231 cells as determined by PI staining assay. (**g**) Effect of CKI-MacOmtOspc subtractive fraction and MOO on cell-cycle in MDA-MB-231 cells as determined by PI staining assay. (**h**) The representative histograms of cell-cycle analysis by the treatments as compared to UT. Statistically significant results shown as (*)P < 0.05 or (**)P < 0.01 or (***)P < 0.001 or (****)P < 0.0001. All data were shown as mean ± SD.

Fractionation and full reconstitution of WRCKI-H (reconstitution using milliQ H_2_O buffered with 10 mM HEPES) or WRCKI-B (reconstitution using buffer/vehicle control) caused no changes in cell viability compared to original CKI (see methods) at either 24- or 48-hour in MDA-MB-231 cells (Supplementary Fig. 2a). As a result, original CKI was used as a basis for comparison for further fractionation experiments. Our results in Supplementary Fig. 2a showed that several cycles of lyophilisation yielded WRCKI mixtures that were indistinguishable from CKI in our bioassay, indicating the complete elimination of the solvents used in HPLC fractionation process. Both CKI and reconstituted WRCKI caused significantly reduced viability compared to untreated (UT) cells at 48-hour after treatment. The MJ subtractive fraction contained a total of nine compounds, including eight previously identified MJ peaks^2^ and adenine, and the MN fraction contained the remaining peaks (Supplementary Fig. 1). MJ had no effect on cell viability, while MN reduced cell viability to the same extent as CKI compared to UT (Fig. 2a). The nine major compounds were individually depleted from CKI and tested as 9 (CKI-1) subtractive fractions, with no significant alterations in cell viability compared to CKI (Fig. 2b). We then assessed the interaction effects of single MJ compounds by adding them back to the MN subtractive fraction. No change in cell viability compared to MN was observed (Supplementary Fig. 2b). Sets of three compounds from the nine major/standard compounds of CKI were depleted to generate 3 (CKI-3) subtractive fractions. The nine reference compounds were allocated into three groups, one of which contained structurally similar compounds (Omt, Ospc, Spc) and two other groups ([Mac, Ade, Tri] and [Nme, Mt, Spr]) that contained structurally different compounds. Of these three groups, only CKI-OmtOspcSpc decreased cell viability (albeit not statistically significantly) compared to CKI after 48 hours (Supplementary Fig. 2c), and none of the sets of three compounds on their own had any effect on cell viability (Supplementary Fig. 2c–e). In order to follow up the suggestion of decreased cell viability from CKI-OmtOspcSpc depletion, we then generated 9 (CKI-2) subtractive fractions based on the CKI-3 subtractive fractions (Table 1). Out of 9 (CKI-2) subtractive fractions (Supplementary Fig. 2), only CKI-OmtOspc significantly decreased cell viability compared to CKI (P < 0.05) (Fig. 2c). We then depleted macrozamin, the only major compound derived from Baituling, together with OmtOspc as CKI-3 (CKI-MacOmtOspc) in order to determine if there was an additional effect when compared to CKI-OmtOspc. CKI-OmtOspc and CKI-MacOmtOspc both decreased cell viability to the same extent (Fig. 2c,d).

While no change in cell viability was found across all CKI-1 treatments, cell-cycle assay was performed to identify more subtle differences. There was no statistically significant difference in phases of the cell-cycle of MDA-MB-231 cells for many of the CKI-1 treatments compared to CKI except for a statistically significant change in G1 phase by CKI-Omt after 48 hours (Fig. 2e). On the other hand, CKI-OmtOspc treatment significantly altered the cell-cycle for MDA-MB-231 cells and induced significantly higher apoptosis from 0.25 mg/ml through 2 mg/ml treatments as compared to CKI at both timepoints (Fig. 2f and Supplementary Fig. 3). CKI-MacOmtOspc treatment also significantly altered the cell-cycle at both timepoints with generally similar effects to CKI-OmtOspc (Fig. 2g,h).

**Figure 3:**
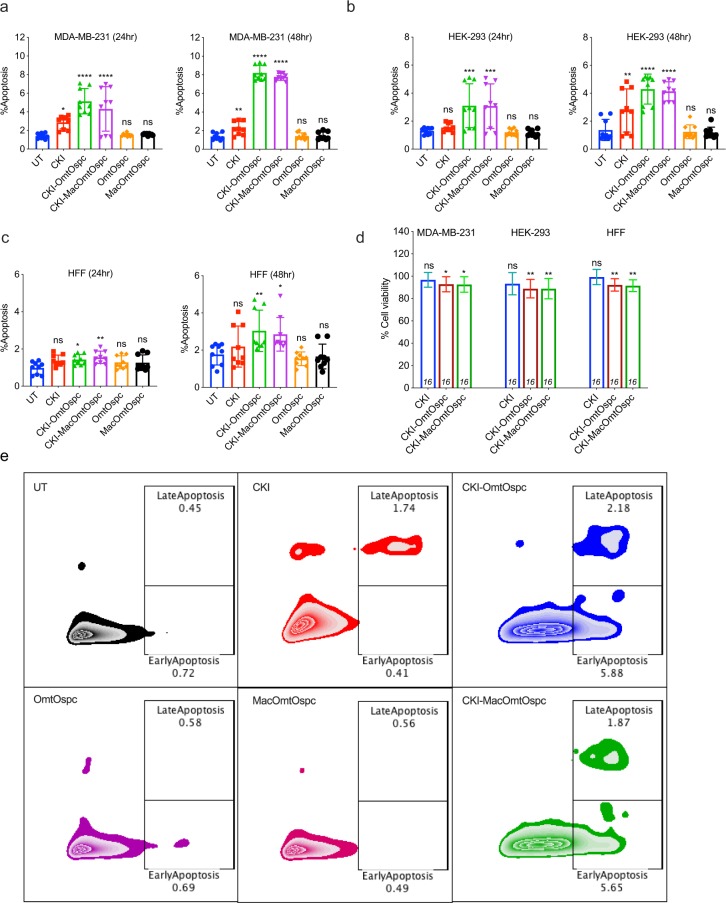
Apoptosis and cytotoxicity assays of subtractive fractions at 24- and 48-hour time point treatments. Apoptotic effect of CKI-OmtOspc, CKI-MacOmtOspc subtractive fractions, OmtOspc and MacOmtOspc in (**a**) MDA-MB-231 cells, (**b**) HEK-293 cells, and (**c**) HFF cells as determined by AnnexinV/PI assay. (**d**) Cytotoxic effect of CKI, CKI-OmtOspc and CKI-MacOmtOspc subtractive fractions was determined using Alamar Blue cytotoxicity assay. All treatments were compared against UT. (**e**) Representative plots of Annexin V and PI staining in MDA-MB-231. Statistically significant results shown as (*)P < 0.05 or (**)P < 0.01 or (***)P < 0.001 or (****)P < 0.0001; ns (not significant). All data were shown as mean ± SD.

Annexin V/PI apoptosis assays were performed using subtractive fractions on MDA-MB-231, HEK-293 (human embryonic kidney cells) and HFF (primary human foreskin fibroblasts) cell lines. While CKI at 2 mg/ml caused increased apoptosis in MDA-MB-231 cells at both 24- and 48-hour after treatment, CKI-OmtOspc and CKI-MacOmtOspc subtractive fractions at concentrations equivalent to CKI 2 mg/ml significantly increased the percentage of apoptotic cells at 24-hour with increasing apoptosis at the 48-hour timepoint, indicating that CKI-OmtOspc and CKI-MacOmtOspc significantly enhanced apoptosis compared to CKI (Fig. 3a,e and Supplementary Fig. 4a). Although CKI did not generally cause apoptosis in HEK-293 or HFF cells, CKI-OmtOspc and CKI-MacOmtOspc subtractive fractions significantly induced apoptosis in HEK-293 cells (***P < 0.001) at 24-hour and 48-hour (****P < 0.0001) and in HFF cells (*P < 0.05 and **P < 0.01) at 24-hour and at 48-hour (**P < 0.01 and *P < 0.05). CKI only induced apoptosis of HEK-293 at 48-hour (**P < 0.01) and showed no significant apoptotic induction in HFF (Fig. 3b,c and Supplementary Fig. 4b,c). These results indicated that the CKI-OmtOspc and CKI-MacOmtOspc subtractive fractions induced apoptosis not only in cancerous cells but also in non-cancerous cell lines. In contrast, no significant apoptosis was triggered by CKI on HFF cells. A small but significant apoptotic induction was observed for HEK-293 cells.

Because of the significantly decreased viability accompanied by increased apoptosis triggered by subtractive fractions, cytotoxicity tests were carried out for all three cell lines using CKI (2 mg/ml) and CKI-OmtOspc and CKI-MacOmtOspc subtractive fractions at concentrations equivalent to CKI 2 mg/ml. CKI-OmtOspc and CKI-MacOmtOspc at equivalent concentration to CKI 2 mg/ml were significantly cytotoxic to both non-cancerous cell lines (Fig. 3d).

Overall, these results indicated that removal of combinations of specific compounds from CKI had unpredictable effects on the ability of CKI to kill cells. While removal of all major compounds from CKI caused no loss of activity and removal of all minor compounds caused total loss of activity, removal of selected major compounds (CKI-OmtOspc) paradoxically caused major, significant increases in the ability of CKI to reduce cell viability and kill cells.

### Differential gene expression

In order to understand the interactions of the components in CKI as a result of depletion, we carried out RNAseq of MDA-MB-231 cells treated with CKI and subtractive fractions. Four out of nine (CKI-1) subtractive fractions, namely CKI-Omt, CKI-Mac, CKI-Tri and CKI-Nme, were selected due to their structural differences, and transcriptomes of cells treated with these fractions for 48-hours were sequenced. CKI-OmtOspc and CKI-MacOmtOspc, OmtOspc, MacOmtOspc and CKI treated cells were sequenced at 24 and 48-hour timepoints. A summary of the samples, number of samples, RNA-Seq sample names, processed sample names and treatments are shown in Supplementary Table 1.

**Figure 4:**
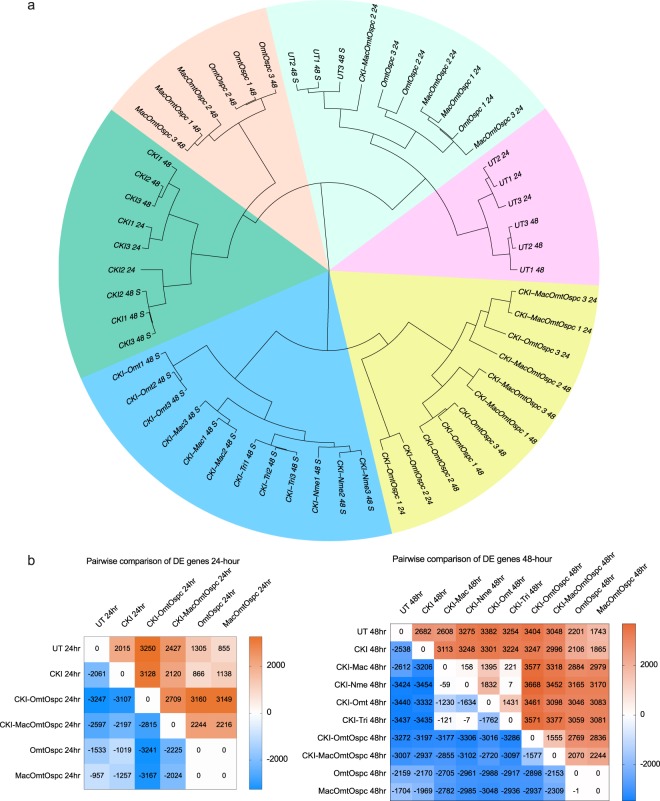
Gene expression clustering and summary of differential gene expression. (**a**) Clustering of treated samples based on gene expression, calculated as transcripts per million using Ward’s hierarchical cluster analysis (Ward.D2) method. Number of DE genes (FDR < 0.05 according to edgeR) associated with each treatment was calculated using pairwise comparison at (**b**) 24-hour and 48-hour timepoints. Treatments were compared column versus row. Up-regulated genes are shown in shades of red and down-regulated genes are shown in shades of blue.

Two batches of RNAseq results were merged in order to compare CKI-1 to CKI-OmtOspc (CKI-2) and CKI-MacOmtOspc (CKI-3) fractions. After removing batch effects with the R package RUV from the merged dataset, CKI treated replicates between the two batches clustered together (Fig. 4a), indicating that gene expression patterns of the samples treated by CKI were similar regardless of the batches. We also examined the correlation between the samples with the same treatments from two batches. The pearson correlation coefficient of untreated samples was 0.95 and of CKI treated samples was 0.94 at 48-hour between the two sequencing batches (Supplementary Fig. 5), indicating a small batch effect. In addition, clear clustering of all 4 (CKI-1) treated samples (Fig. 4a and Supplementary Figs 6–9), showed that these replicates share comparable gene expression patterns. Likewise, OmtOspc and MacOmtOspc groups and CKI-OmtOspc and CKI-MacOmtOspc groups showed similar changes in gene expression, except for one replicate (CKI-MacOmtOspc, 24-hour) that clustered with UT, OmtOspc and MacOmtOspc.

The number of DE genes associated with each treatment was calculated using pairwise comparative analysis. CKI treatment was used as a baseline to compare all other treatments in order to emphasize the effect of depleted compounds and CKI treatment was compared to UT.

There were thousands of upregulated and downregulated genes at 24- and 48-hours in most pairwise comparisons (Fig. 4b). However DE genes between OmtOspc and MacOmtOspc treatments were not observed and there were almost no DE genes between CKI-Mac, CKI-Nme and CKI-Tri treatments (Fig. 4b) indicating that these three subtractive fractions had very similar effects on gene expression.

When we compared the DE genes found between treatments, there were a large number of DE genes (~71.3%) shared between all four (CKI-1) treatments (Supplementary Fig. 10 and Supplementary Table 2). A similar number of shared DE genes (~24.6%) between four (CKI-1), OmtOspc and MacOmtOspc and between four (CKI-1), CKI-OmtOspc and CKI- MacOmtOspc as compared to CKI at 48-hour indicated that gene expression patterns from CKI-1 treatments were mostly different from CKI-OmtOspc, CKI-MacOmtOspc, OmtOspc and MacOmtOspc treated cells. 55% of the DE genes between UT, OmtOspc and MacOmtOspc were shared. When the four (CKI-1) treatments were compared to CKI treatment, 42.8% of DE genes were shared, and when CKI-OmtOspc and CKI- MacOmtOspc treatments were compared to CKI, 50.1% DE genes were shared, indicating that CKI-OmtOspc and CKI-MacOmtOspc treatments appeared to be more similar to CKI than CKI-1 treatments.

The overall levels of similarity in DE genes were as follows: 1) All CKI-1 treatments had approximately 70% similar gene expression patterns, 2) OmtOspc and MacOmtOspc treatments were approximately 50% similar to UT and 33% similar to CKI-1 treatments, 3) gene expression patterns between CKI-1, CKI-OmtOspc and CKI-MacOmtOspc were approximately 37% similar.

### Gene ontology and pathway annotation of DE genes

**Figure 5:**
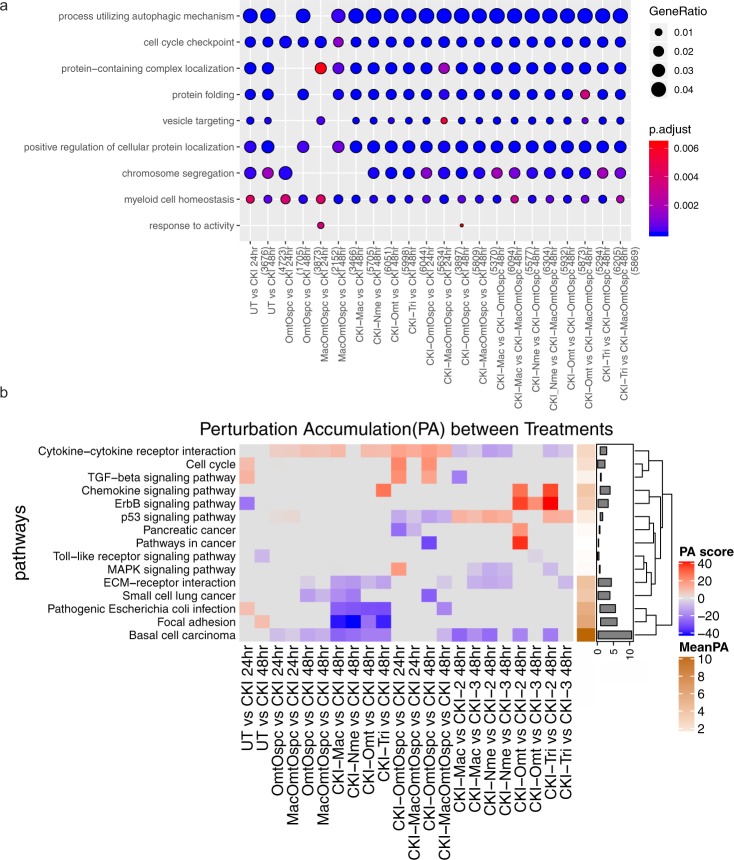
Over-representation analysis of GO functional annotation and KEGG pathway perturbation analysis. (**a**) Over-represented GO terms (Biological Process at level 3) for DE genes identified from comparison of subtractive fraction-treated cells against CKI treatment in order to show the relative change from depleting compounds. Gene ratio of each term calculated from clusterProfiler was plotted based on the adjusted p-values. The top 5 most statistically significantly over-represented categories of GO terms were plotted by default. Colour gradient of adjusted p-values ranging from red to blue in order of increasing p-values (high to low significance). Number of identified genes in each treatment (numbers in parentheses) were shown in the bottom and the sizes of the dots correspond to the ratio of genes out of all significant DE gene from each treatment involved in the particular terms. (**b**) Identification of significantly perturbed pathways using SPIA (pG < 0.05) analysis. Eighty-nine significantly perturbed pathways from twenty-two comparisons were found (Supplementary Fig. 10). Only the 15 pathways most obviously linked to our phenotypes of cell viability, cell-cycle and apoptosis were shown here. Positive (overall increase in gene expression for pathway) and negative (overall decrease in gene expression for pathway) perturbation accumulation values of the pathways were shown in red and blue, respectively. Mean perturbation values of each pathway were shown in bar plot.

DE genes were analysed for over-representation in our data sets with respect to biological function using Gene Ontology (GO) annotation. We looked for shared DE genes between treatments and identified over-represented functional terms in these shared genes. The only common function enriched across all comparisons was for “cell-cycle checkpoint” (Fig. 5a). This confirmed earlier results^16^ and was consistent with the phenotype data for CKI.

### Subtracted fractionation altered pathways

**Figure 6:**
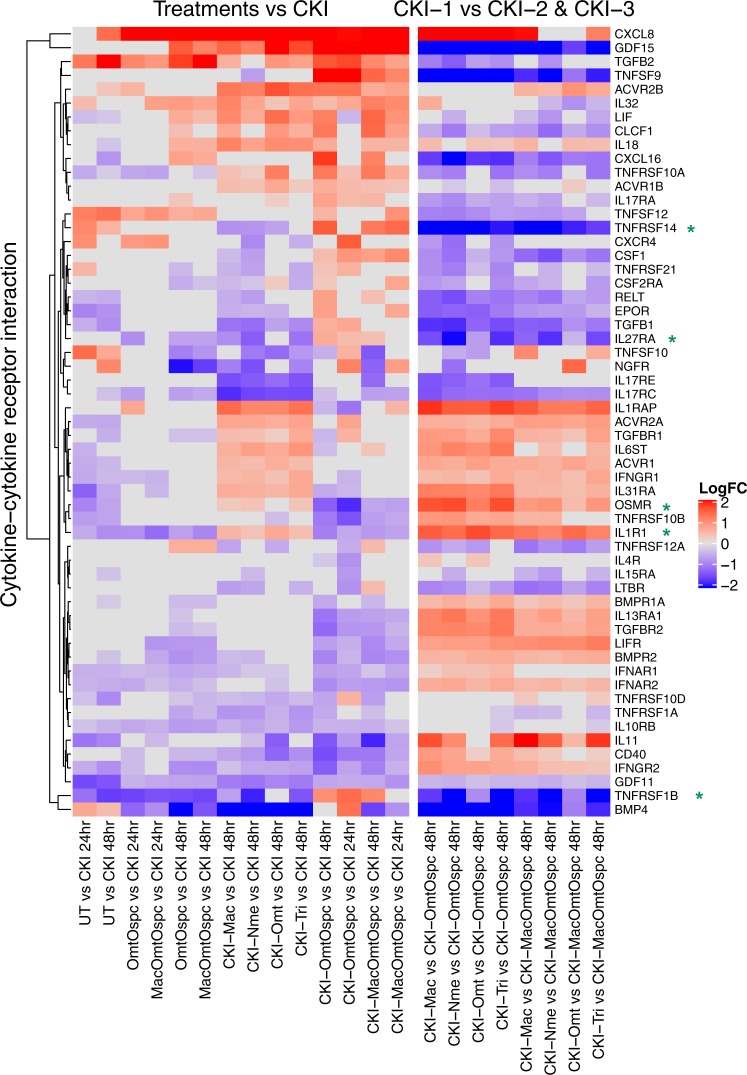
Differential gene expression profiles of all treatments for Cytokine-Cytokine Receptor pathway. The left panel shows comparison of subtractive fraction treated cells against CKI treatment and the right panel shows comparison of single compound subtractive fraction treated cells against the treatments for two and three compound subtractive fractions. Asterisks in green show a subset of genes that had opposite changes in gene expression across treatments.

**Figure 7:**
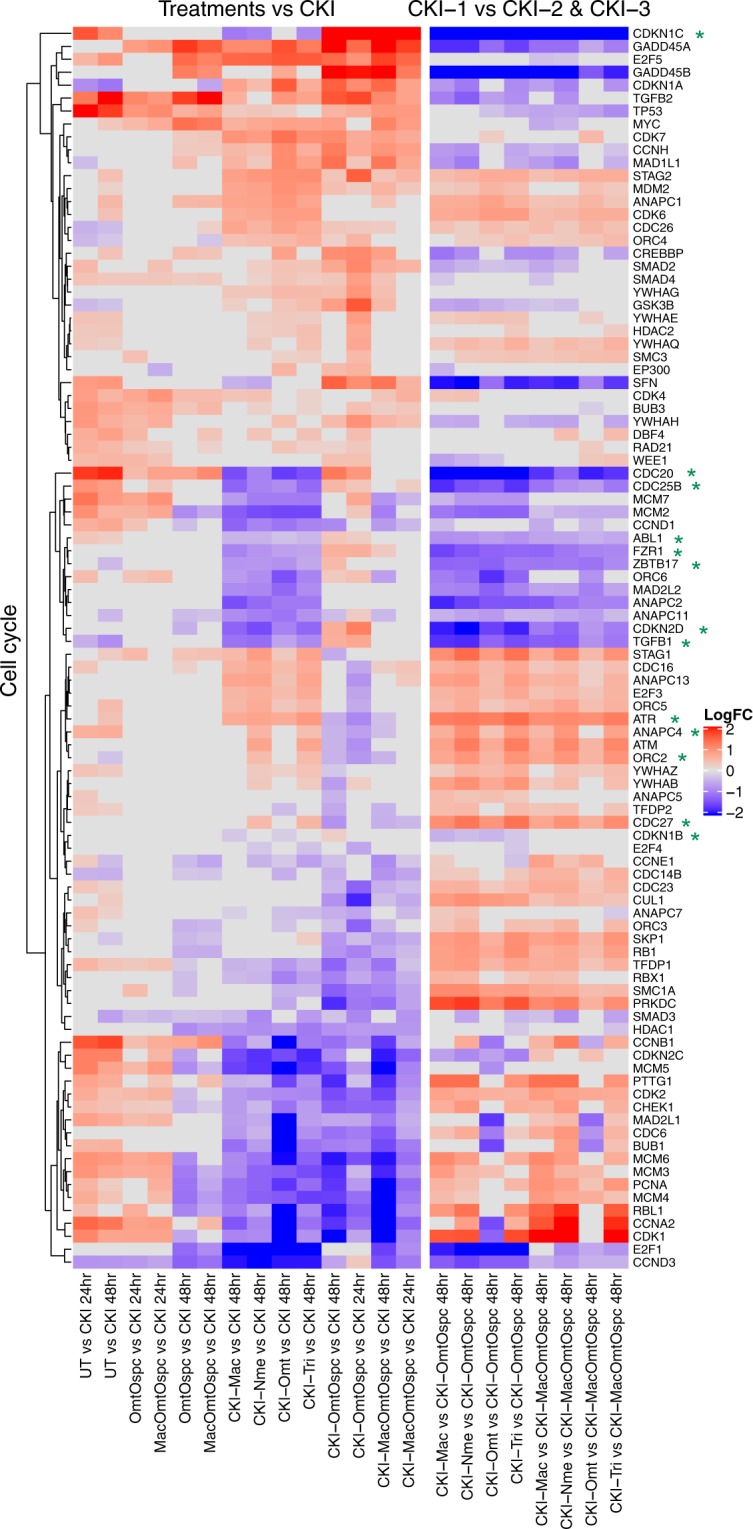
Differential gene expression profiles of all treatments for Cell-Cycle pathway. The left panel shows comparison of subtractive fraction treated cells against CKI treatment and the right panel shows comparison of single compound subtractive fraction treated cells against the treatments for two and three compound subtractive fractions. Asterisks in green show a subset of genes that have opposite changes in gene expression across treatments.

We also performed pathway-based analysis to look for pathway level perturbation by comparing DE genes within Kyoto Encyclopedia of Genes and Genomes (KEGG) pathways between treatments. We used Signaling Pathway Impact Analysis (SPIA) to identify pathways with statistically significant perturbation values expected to alter pathway flux. We identified 86 pathways (Supplementary Fig. 11) with statistically significant (P < 0.05) perturbations of gene expression and of these, 15 pathways were most obviously linked to our phenotypes of cell viability, cell-cycle and apoptosis (Fig. 5b). By comparing the pathway gene expression global perturbation scores (pG) between treatments, three specific observations could be made: (1) CKI-1 fractional deletions vs CKI had significant effects on flux in some pathways without phenotypic effects, (2) CKI-OmtOspc vs CKI, which had a pronounced phenotypic effect at both 24- and 48-hours, had a significant effect on reducing estimated pathway flux for Cytokine-Cytokine Receptor, Cell-Cycle and TGF-Beta signaling pathways (Figs 6 and 7 and Supplementary Figs 12–21), (3) comparison of CKI-1 fractional deletions vs fractional deletions of CKI-OmtOspc/CKI-MacOmtOspc showed consistent pathway perturbations for Cytokine-Cytokine Receptor and p53 signaling pathways. On this basis, we inferred that different major compounds could be deleted with very similar effects, indicating that they may have similar targets. In contrast, deleting Omt and Ospc simultaneously caused a significant shift in phenotype and was accompanied by specific perturbations in pathways that regulate inflammation, cell-cycle and apoptosis. The combined deletion of Omt and Ospc had a synergistic effect on cell viability, cell-cycle and apoptosis and a synergistic effect on gene expression, consistent with the observed changes in pathway specific perturbation of gene expression. Because this double compound deletion potentiated the cell killing effect of CKI we hypothesised that the compounds in CKI have multiple targets leading to a phenotypic effect that reflects the integration of stimulation and inhibition across all those targets. Removal of Omt and Ospc alters the balance of stimulation and inhibition leading to an integrated effect for the remaining compounds in the mixture that caused more cell death than CKI.

More detailed examination of some of these interactions within significantly perturbed pathways highlighted the gene-specific changes in expression for some key regulators of inflammation and the cell-cycle. Most effects on gene expression from deletion of single versus two compounds were similar, suggesting that the enhanced cell killing by CKI-OmtOspc was due to additive effects of the compound deletions. However, by comparing differences in pairwise comparisons between treatments at the gene level within the Cytokine-Cytokine Receptor Interaction and Cell-Cycle pathways we identified a subset of genes that had opposite changes in gene expression when comparing single compound deletions to CKI-OmtOspc deletion. In the Cytokine-Cytokine Receptor Interaction pathway (Fig. 6 and Supplementary Figs 12–14), these genes are *IL1-R1* (Interleukin-1 Receptor), *IL-27RA* (Interleukin-27 Receptor alpha subunit), *TNFRSF1B* (Tumor Necrosis Factor Receptor Superfamily Member 1B), *TNFRSF14* (Tumor Necrosis Factor Receptor Superfamily, Member 14) and *OSMR* (Oncostatin M Receptor/IL-31 Receptor Subunit Beta), and they all transduce inflammatory ligand signals to the NF_k_B pathway and/or the apoptosis pathway. In the Cell-Cycle pathway (Fig. 7 and Supplementary Figs 15–17), these genes are *CDKN1C* (Cyclin-Dependent Kinase Inhibitor 1C (P57, Kip2)), *CDC25B* (Cell Division Cycle 25B), *ATR* (ATR Serine/Threonine Kinase), *CDKN1B* (Cyclin-Dependent Kinase Inhibitor 1B (P27, Kip1)), *CDKN2D* (Cyclin-Dependent Kinase Inhibitor 2D (P19, Inhibits CDK4)), *TGFB1* (Transforming Growth Factor Beta 1), *FZR1* (Fizzy And Cell Division Cycle 20 Related 1), *CDC20* (Cell Division Cycle 20), *CDC27* (Cell Division Cycle 27), *ORC2* (Origin Recognition Complex Subunit 2), *ANAPC4* (Anaphase Promoting Complex Subunit 4), *ZBTB17* (Zinc Finger And BTB Domain Containing 17) and*, ABL1* (ABL Proto-Oncogene 1, Non-Receptor Tyrosine Kinase). The opposite changes in gene expression stimulated by CKI-OmtOspc compared to CKI-1 subfractions provide support for the idea that multiple major compounds can have similar effects on specific genes but that the combination of Omt and Ospc can have synergistic and opposite effects on those same genes. This means that multiple compounds with overlapping targets (based on their structural similarities) can either reinforce a single outcome or exhibit unpredictable and opposite effects when combined.

Overall our results support the concept of multi-compound/multi-target interactions for plant extract-based drugs that contain many plant secondary metabolites. Biological effects of complex plant extracts may result from interactions of multiple compounds, with negligible effects from single compounds alone. This has implications for how we assess the functional evidence for such extracts.

## Discussion

Previous studies have demonstrated that CKI can alter the cell-cycle, induce apoptosis and reduce proliferation and migration in various cancer cell lines^6,16–18^. CKI also killed leukaemia cells via the Prdxs/ROS/Trx1 signalling pathway in an acute myeloid leukaemia patient-derived xenograft model and caused cell-cycle arrest in U937 leukaemia-derived cells^19^. Cell-cycle arrest by CKI at checkpoints is correlated with the induction of double strand breaks by CKI treatment^20^. In contrast to our experiments reported above, oxymatrine was previously shown to arrest the cell-cycle and induce apoptosis in human glioblastoma cells through EGFR/PI3K/Akt/mTOR signaling pathway^21^ and inhibit the proliferation of laryngeal squamous cell carcinoma Hep-2 cells^22^. As shown in this report, oxymatrine or oxysophocarpine or combined OmtOspc treatment caused no significant change in cell viability, the cell-cycle or apoptosis, in agreement with prior work that showed oxymatrine and oxysophocarpine exerting no significant effect on apoptosis, cell-cycle or cell proliferation in HCT116 human colon cancer cells^23^.

The paradoxical result that removal of OmtOspc caused a striking increase in apoptosis is most simply explained by a model based on integrating effects of multiple compounds on many targets. The interactions between compounds in the mixture can be synergistic and antagonistic such that if two compounds are removed that have a synergistic effect that is antagonistic to the remainder of the mixture, the resulting depleted mixture will be dis-inhibited compared to CKI. This is illustrated by our studies and others that show single compounds alone had no or little effect compared to CKI. For instance, while CKI treatment resulted in increased DNA double strand breaks and affected the cell-cycle resulting in decreased cancer cell proliferation, oxymatrine alone exhibited only a small effect in the same assay^20^. Gao and colleagues also reported that oxysophocarpine at 4 mg/ml had no effect, oxymatrine at 4 mg/ml (*P < 0.05) and CKI at 2 mg/ml (***P < 0.001) significantly reduced the proliferation of hepatocellular carcinoma SMMC-7721 cells *in vitro*^6^. Although significant inhibition of proliferation by oxymatrine occurred, the concentration used in this experiment was ~8 times higher than that of oxymatrine in 2 mg/ml of CKI. These studies agree with our experimental outcomes that oxymatrine and oxysophocarpine individually had no or little effect compared to CKI treatment.

At the level of gene expression in our study, GO analysis indicated that genes for “cell-cycle checkpoint” were significantly enriched in cells treated with all fractionated mixtures or mixtures of Omt and Ospc. Consistent with other studies, our results also demonstrated that these compounds had little or no phenotypic effect on their own, but that when both were deleted, the remaining compounds unexpectedly had significantly greater effects on phenotype and gene expression. When examined in the context of specific pathways, treatment with OmtOspc or CKI-OmtOspc which had strikingly different effects on phenotype, had similar effects on the perturbation of the “Cytokine-Cytokine Receptor Interaction” pathway, the most commonly perturbed pathway seen in our analysis that interestingly did not show up when comparing UT to CKI. This is consistent with previous work showing that CKI induced cytokines IL4 and IL10 in cancer patients with acute leukaemia^24^ and administration of CKI significantly increased the levels of IgA, IgG, IgM, IL2, IL4 and IL10, and decreased the levels of IL6 and TNF-α in rats with induced gastric cancer^25^. In contrast to this observation, IL4 and IL10 levels were significantly decreased in transgenic mice treated with oxymatrine at a dose of 200 mg/kg^26^. In our experiment, we also observed that while CKI and many of the depleted fractions had significant effects on the genes in the “Cytokine-Cytokine Receptor Interaction” pathway, OmtOspc and MacOmtOspc had little effect on the genes in that pathway. The observation that many genes in the “Cytokine-Cytokine Receptor Interaction” pathway were not affected by OmtOspc and MacOmtOspc compared to deletion fractions confirmed that removal of compounds rather than treatment with single or a few compounds can be more informative of the role and significance of individual compounds as part of mixtures/extracts.

In summary, our approach allowed the identification of both synergistic and antagonistic interactions within the drug mixture. Viewed as a network where the compounds and the targets are nodes and the interactions between compounds and targets, and between targets are edges, it is clear that the edges (interactions) determine the overall effect of the compound mixture. By removing one or two compounds from a mixture, we can potentially perturb the target network(s) to either reduce the effect of the mixture for some outcome or potentiate it for another. We believe this approach may be of general use for the study of herbal medicines/extracts, avoiding failures that stem from exclusive reliance on the identification of a single compound that accounts for most of the biological activity in mixtures.

## Methods

### Cell lines

MDA-MB-231 cells were purchased from the American Type Culture Collection (ATCC, VA, USA). HEK-293 and HFF were kindly provided by Prof. Andrea Yool (Medical School, University of Adelaide). Cells were cultured in Dulbecco’s Modified Eagle’s Medium (Thermo Fisher Scientific) with 10% Fetal bovine serum (Thermo Fisher Scientific) at 37 °C with 5% CO_2_.

### Compound fractionation by HPLC

CKI (Batch No: 20170322, total alkaloid concentration of 26.5 mg/ml) was provided by Zhendong Pharmaceutical Co.Ltd (China). HPLC separation for CKI was achieved using a Shimadzu HPLC instrument (Japan) equipped with a photodiode-array UV-Vis detector with preparative C_18_ column (5 µm, 250 × 10 mm) (CA, USA). The following mobile phase was used to fractionate the CKI mixture: 0.01 M ammonium acetate (adjusted to pH 8.0, solvent A) and acetonitrile + 0.09% trifluoroacetic acid (solvent B). The flow rate was 2 ml/min and a linear gradient was used as follows: 0 min, 100% A; 60 min, 65% A; 70 min, 100% A. The chromatogram was recorded from 200 nm to 280 nm, with monitoring at 215 nm. Samples were frozen and lyophilised using a Christ Alpha 1-2 LD lyophiliser (Martin Christ Gefriertrocknungsanlagen GmbH, Germany). CKI 1 ml was fractionated on HPLC column and whole reconstituted CKI (WRCKI) was generated by collecting all fractions. Three rounds of lyophilisation were performed to remove the solvents used during the HPLC fractionation process and final reconstitution for all samples was carried out using vehicle control buffer [MilliQ H_2_O containing 0.25% Tween 80 and 10 mM HEPES (Gibco, Life technologies, USA)] or MilliQ H_2_O buffered with 10 mM HEPES and adjusted to pH 6.8–7.0. For example, WRCKI-H was resuspended with equivalent volume 1 ml using MilliQ H_2_O buffered with 10 mM HEPES and adjusted to pH 6.8–7.0.

CKI was then processed to deplete single (CKI-1), double (CKI-2) and triple (CKI-3) compounds using HPLC by standardizing using nine compounds, namely Oxymatrine (Omt), Oxysophocarpine (Ospc), N-methylcytisine (Nme), Matrine (Mt), Sophocarpine (Spc), Trifolirhizin (Tri), Adenine (Ade), Sophoridine (Spr) (Beina Biotechnology Institute Co., Ltd, China), and Macrozamin (Mac) (Zhendong Pharmaceutical Co.Ltd, China) which were previously reported to be found in CKI. In single compound depletions (CKI-1), CKI-Omt for example, Oxymatrine peak between 26–28 minutes was removed and the remaining HPLC fractions were collected, lyophilised three times and reconstituted for further experiments. This procedure was applied to all CKI-1, CKI-2 and CKI-3 fractions. HPLC fractionation separated Minor (MN) and Major (MJ) peaks to determine the principal and secondary components. The MJ mixture contained the nine standard compounds mentioned above and MN contained the remaining CKI components. In addition, nine CKI-1 fractional deletions, nine CKI-2 fractional deletions and three CKI-3 fractional deletions were produced.

### Identification of reconstituted mixtures by liquid chromatography/mass spectrometry (LC-MS/MS)

Agilent 6230 TOF mass spectrometer was used to determine the concentration of the known compounds from the CKI and reconstituted CKI-OmtOspc and CKI-MacOmtOspc mixtures. 10 µl sample was injected with a flow rate of 0.8 ml/min, a gradient program of 0 min, 100% A; 25 min, 40% B; 35 min, 60% B; and solvents MilliQ H_2_O + 0.1% formic acid (solvent A) and acetonitrile + 0.1% formic acid (solvent B). The column used was C_18_ (5 μm, 150 × 4.6 mm, Diamosnsil, Dkimatech, China). The recovered contents of the samples were measured by spike-in compound cytosine. Gas phase ions were generated with an electrospray source, with key instrument parameters: gas temperature, 325; sheath gas temperature, 350; vCap, 3500; fragmentor, 175; acquisition range (m/z) 60–17000. Calibration curves for nine standard compounds containing various concentrations were shown in Supplementary Data.

### Cell viability assay

2,3-bis-(2-methoxy-4-nitro-5-sulfophenyl)-2H-tetrazolium-5-carboxanilide (XTT) and N-methyl dibenzopyrazine methyl sulfate (PMS) (50: 1, Sigma-Aldrich, St. Louis, MO, USA) assay was used to assess cell viability as described in Qu *et al*.^16^. Briefly, 8,000 cells in 50 µl of medium were plated in 96-well trays overnight prior to drug treatments in triplicate. On the following day, CKI (at a final concentration of 0.25, 0.5, 1 and 2 mg/ml total alkaloid) and fractionated mixtures (equivalent dilutions of CKI) were added. For example, dilution 1/13.25 is equal to CKI 2 mg/ml and equivalent 1/13.25 dilution was performed for all fractionated mixtures to achieve 2 mg/ml equivalent dilutions of CKI. Cells were subsequently treated with 50 µl of drug mixtures to provide final concentrations of 0.25, 0.5, 1 and 2 mg/ml of total alkaloids in 100 µl. Cell viability was then measured at 24- and 48-hour after drug treatment by the addition of 50 µl of XTT:PMS mixture (50: 1 ratio). An equal volume of medium and treating agents plus XTT: PMS was used to subtract the background optical density. The absorbance of each well was recorded using a Biotrack II microplate reader at 492 nm. The experiments were performed twice by each of three different operators and each experiment had three technical replicates.

### Annexin V/PI apoptosis assay

Apoptosis resulting from treatment was determined using an Annexin V-FITC apoptosis detection kit (Thermofisher Scientific) according to the manufacturer’s protocol. Briefly, 4 × 10^5^ cells were seeded in 6-well plates in triplicate overnight prior to treatment. On the following day, cells were treated with the agents as described for 24 and 48 hours. Data were acquired with a BD LSR Fortessa X20 (BD BioSciences, NJ, USA) flow cytometer, and FlowJo software (TreeStar Inc., OR, USA) was used to analyse the acquired data and produce percent apoptosis values.

### Cell-cycle assay

Cell culture and drug treatments were performed as described above for cell-cycle analysis. A Propidium Iodide (PI) staining protocol^27^ was used to detect the changes in cell-cycle as a result of treatment after 24 and 48 hours. The characteristics of stained cells were measured using a BD LSR Fortessa flow cytometer, and acquired data were analysed using FlowJo software. The experiments were performed twice by each of three different operators and each experiment had three technical replicates.

### Cytotoxicity assay

Cells were seeded in 96-well plates at a density of 2.5 × 10^3^ cells per well in triplicate. CKI and fractionated mixtures at final concentrations of 1 mg/ml and 2 mg/ml were added to each well and after 24 hours of incubation and viable cells were measured using the Alamar Blue assay (Thermo Fisher Scientific). 5 µM of Mercuric chloride (Sigma-Aldrich) was used as a positive control and wells without cells were set as a negative control in the same plate. The experiments were performed twice and each experiment had three technical replicates.

### Sample preparation and RNA sequencing

Cells were plated in 6-well plates with a density of 2 × 10^5^ cells/well overnight prior to drug treatments. On the following day, CKI (at a final concentration of 2 mg/ml) and fractionated mixtures (equivalent dilutions of CKI) were added. Two batches of samples were prepared. In the first batch, cells were treated with CKI, CKI-OmtOspc and CKI-MacOmtOspc at 24- and 48-hour timepoints in triplicates and in the second batch, cells were treated with CKI, CKI-Mac, CKI-Nme, CKI-Omt and CKI-Tri at 48-hour timepoint in triplicates along with 3 UT replicates in both batches. Total RNA was isolated by using PureLink^TM^ RNA mini kit (Thermo Fisher Scientific) according to the manufacturer’s instructions and the quantity and quality of RNA samples were determined using a Bioanalyzer at the Cancer Genome Facility of the Australian Cancer Research Foundation (Australia). RNA samples with RNA integrity number (RINs) > 7.0 were sent to be sequenced at Novogene (China). Briefly, after QC were performed, mRNA was isolated using oligo (dT) beads and randomly fragmented by adding fragmentation buffer, followed by cDNA synthesis primed with random hexamers. Next, a custom second-strand synthesis buffer (Illumina), dNTPs, RNase H and DNA polymerase I were added for second-strand synthesis. After end repair, barcode ligation and sequencing adaptor ligation, the double-stranded cDNA library was size selected and PCR amplified. Sequencing was carried out on an Illumina HiSeq X platform with paired-end 150 bp reads.

### Transcriptome data processing

FastQC (v0.11.4, Babraham Bioinformatics) was used to check the quality of raw reads before proceeding with downstream analysis. Trim_galore (v0.3.7, Babraham Bioinformatics) with the parameters:–stringency 5 –paired –fastqc_args was used to trim adaptors and low-quality sequences. STAR (v2.5.3a) was then applied to align the trimmed reads to the reference genome (hg19, UCSC) with the parameters:–outSAMstrandField intronMotif–outSAMattributes All–outFilterMismatchNmax 10–seedSearchStartLmax 30–outSAMtype BAM SortedByCoordinate^28^. Then, subread (v1.5.2) was used to generate read counts data with the following parameters featureCounts -p -t exon -g gene_id^29^. Significantly differentially expressed genes between all treatments and CKI were analysed and selected using edgeR (v3.22.3) with false discovery rate (FDR) < 0.05^30^.

Removal of unwanted variance (RUVs) package in R was applied to two different batches of transcriptome datasets to eliminate batch variance^31^. Pearson correlation coefficient between samples with the same treatments (CKI and UT at 48-hour) of two batches were analysed to confirm the variances were minimal between two batches. Three replicates of UT from each batch (UT 48; Batch1 and UT 48S; Batch2) and CKI treated samples from each batch (CKI 48; Batch1 and CKI 48 S; Batch2) at 48-hour time point were combined in order for the two batches of RNA-Seq samples to be processed in one single analysis. CKI treatment was used as a baseline to compare with all other treatments in order to emphasize the effect of depleted compounds. Analyses of Phylogenetics and Evolution (APE) was used to cluster the treatments^32^ followed by RUV. GO and KEGG over-representation analyses were performed using clusterProfiler with the parameters ont = “BP”(Biological Process), pAdjustMethod = “BH”, pvalueCutoff = 0.01, and qvalueCutoff = 0.05^33,34^. Signalling Pathway Impact Analysis (SPIA) was carried out to identify the commonly perturbed pathways within the treatments using the SPIA R package^35^. KEGG database used is the public domain version (KEGG data for SPIA analysis was downloaded from KEGG’s website on: 09/07/2012) that is released as part of SPIA. Significantly perturbed pathways were visualised using Pathview package in R^36^.

### Statistical analysis

Statistical analyses were carried out using GraphPad Prism 8.0 (GraphPad Software Inc., CA, USA). Student’s t‐test or ANOVA (one‐way or two‐way) was used when there were two or three groups to compare respectively. Post hoc “Bonferroni’s multiple comparisons test” was performed when ANOVA results were significant. Statistically significant results were represented as (*)P < 0.05 or (**)P < 0.01 or (***)P < 0.001 or (****)P < 0.0001; ns (not significant). All data were shown as mean ± standard deviation (SD).

### Declaration of transparency and scientific rigour

This declaration acknowledges that this paper adheres to the principles for transparent reporting and scientific rigour of preclinical research recommended by funding agencies, publishers and other organisations engaged with supporting research.

## Supplementary information

Supplementary Information

Dataset 1

## Data Availability

All raw data and the processed data (gene row count (logCPM)) obtained in this study were deposited in the National Center for Biotechnology Information (NCBI) Gene Expression Omnibus (GEO) with the Superseries accession number GSE125743, available at https://www.ncbi.nlm.nih.gov/geo/query/acc.cgi?acc=GSE125743.
