# Supplementary material for: Fractional Deletion of Compound Kushen Injection Indicates Cytokine Signaling Pathways are Critical for its Perturbation of the Cell Cycle
Source: Sci Rep. 2019 Oct 2;9:14200. doi: 10.1038/s41598-019-50271-4 (PMC6775143; doi:10.1038/s41598-019-50271-4)
Supplement: Supplementary file 1 — Supplementary Information [file 41598_2019_50271_MOESM1_ESM.pdf]

Fractional Deletion of Compound Kushen Injection Indicates Cytokine Signaling Pathways are Critical for its Perturbation of the Cell Cycle

Running title: Fractional Deletion of Compound Kushen Injection

Keywords: Sophora flavescens, Heterosmilax chinensis, alkaloid, matrine

Aung TN[1]†, Nourmohammadi S[2]†, Qu Z [1], Harata-Lee Y [1], Cui J[1], Shen HY[1], Yool AJ[2], Pukala T[3], Hong Du[4], Kortschak RD[1], Wei W[5] and Adelson DL*[1].

[1] Department of Molecular and Biomedical Science, School of Biological Sciences, University of Adelaide, Adelaide, South Australia, 5005.

[2] Adelaide Medical School, University of Adelaide, Adelaide, South Australia, 5005.

[3] School of Physical Sciences, University of Adelaide, Adelaide, South Australia, 5005.

[4] School of Chinese Materia Medica, Beijing University of Chinese Medicine, Beijing, 100029, P.R.China.

[5] Beijing Zhendong Guangming Pharmaceutical Research Institute, Shanxi – Zhendong Pharmaceutical Co Ltd, Beijing, P.R. China.

†Co-1st authors: Aung TN and Nourmohammadi S

Thazin Nwe Aung ORCID ID: 0000-0003-4150-0426

Saeed Nourmohammadi ORCID ID: 0000-0002-9469-2874

***Corresponding author: Adelson DL**

Department of Molecular and Biomedical Science, School of Biological Sciences, University of Adelaide, Adelaide, South Australia, 5005.

Telephone: +61 8 8303 7555, Email: david.adelson@adelaide.edu.au

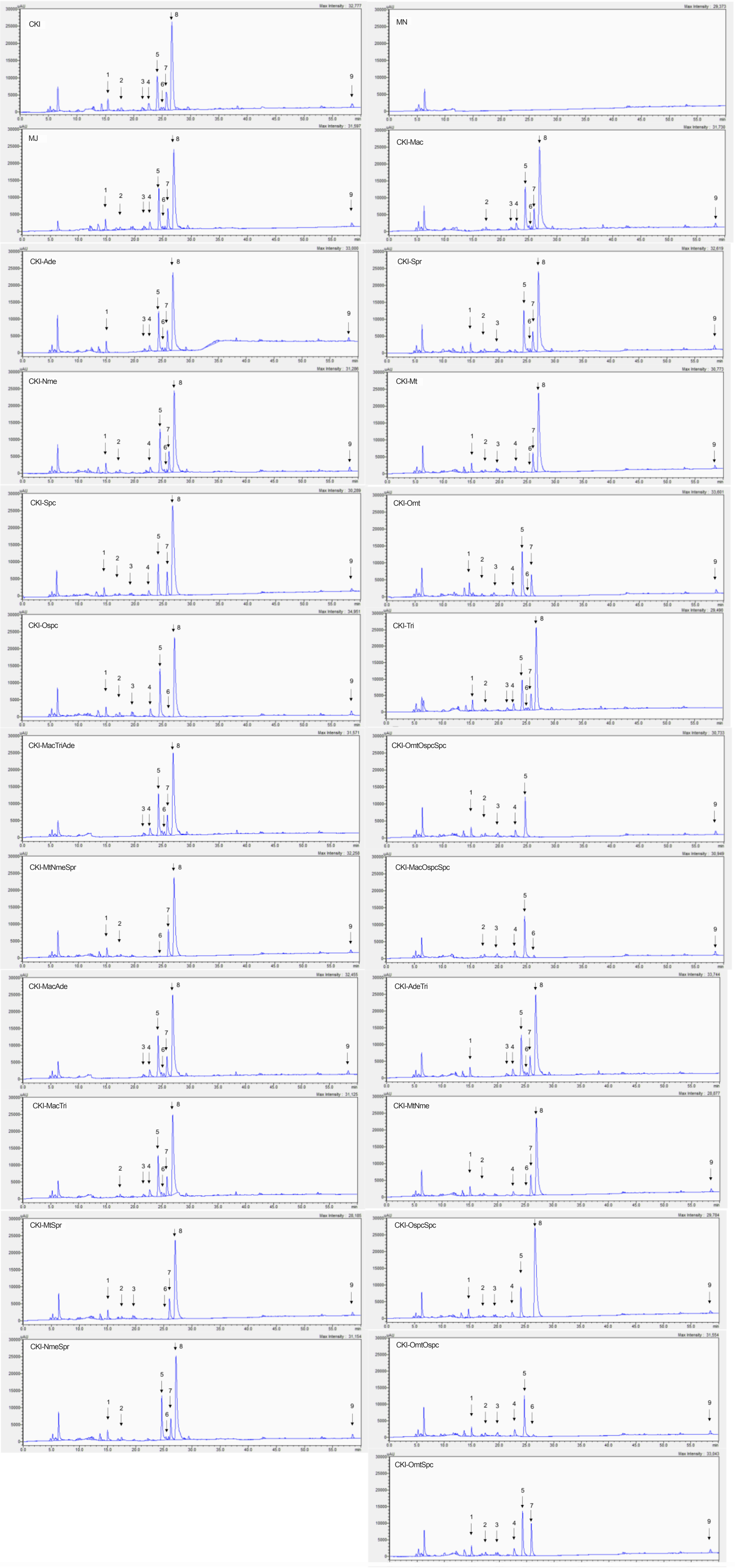
CKI
MN
MJ
CKI-Mac
CKI-Ade
CKI-Spr
CKI-Nme
CKI-Mt
CKI-Spc
CKI-Omt
CKI-Ospc
CKI-Tri
CKI-MacTriAde
CKI-OmtOspcSpc
CKI-MtNmeSpr
CKI-MacOspcSpc
CKI-MacAde
CKI-AdeTri
CKI-MacTri
CKI-MtNme
CKI-MtSpr
CKI-OspcSpc
CKI-NmeSpr
CKI-OmtOspc
CKI-OmtSpc

Supplementary Fig. 1: HPLC profiles of 25 mixtures including CKI, MJ, MN, 9 (CKI-1), 4 (CKI-3), and 6 (CKI-2). MJ shows the HPLC profile of the 9 purified and reconstituted major peaks (MJ) demonstrating nine major compounds. MN shows the HPLC profile of reconstituted fractions not containing the 9 major compounds (MN) showing the remaining peaks with no remaining major compounds. Numbers represent compounds 1: Mac, 2: Ade, 3: Nme, 4: Spr, 5: Mt, 6: Spc, 7: Ospc, 8: Omt and 9: Tri. 50 µl of the samples at 1 mg/ml concentration was injected through the semi-preparative column to achieve the profiles.

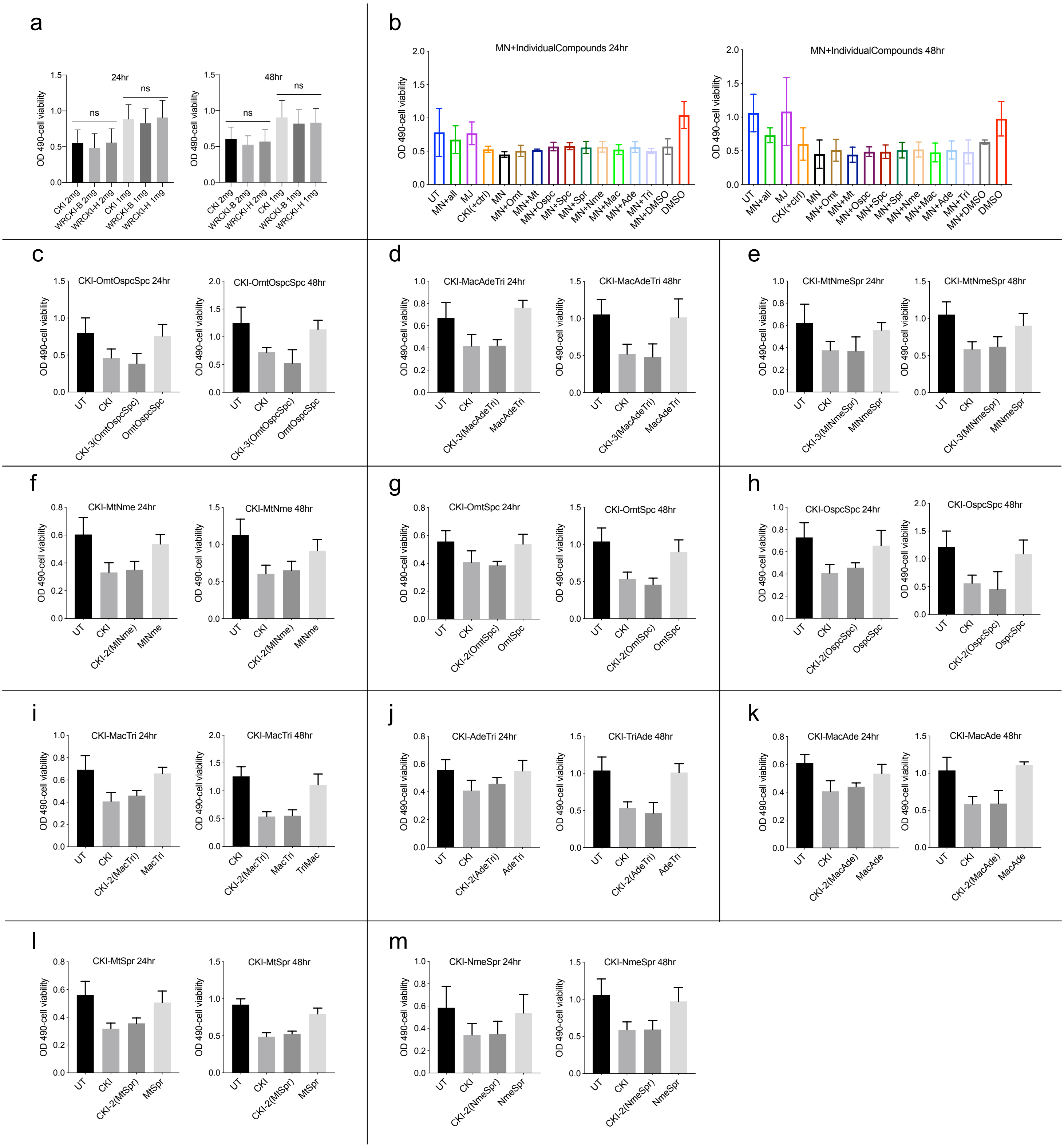

Supplementary Fig. 2: XTT cell viability assays of subtractive fractions in MDA-MB-231 cells at 24- and 48-hour timepoints treated with 1 mg/ml or 2 mg/ml of CKI and 2 mg/ml equivalent concentrations of all other treating agents. (a) Suppression of cell viability from the following treatments: CKI, WRCKI-B (whole reconstituted CKI in buffer/vehicle control), and WRCKI-H (whole reconstituted CKI in milliQ H_2O buffered with 10 mM HEPES). Comparisons were performed between treatments (CKI vs WRCKI-B and CKI vs WRCKI-H). (b) Assessment of the interaction effects of single MJ compounds by the addition to the MN subtractive fraction. Single major compounds were dissolved in either MilliQ H_2O or Dimethyl sulfoxide. Effect of subtractive fractions (c) CKI-OmtOspcSpc, (d) CKI-MacAdeTri, (e) CKI-MtNmeSpr, (f) CKI-MtNme, (g) CKI-OmtSpc, (h) CKI-OspcSpc, (i) CKI-MacTri, (j) CKI-AdeTri, (k) CKI-MacAde, (l) CKI- MtSpr, (m) CKI-NmeSpr. Statistically significant results shown as P < 0.05 (*) or P < 0.01 (**) P < 0.001 (***), or P < 0.0001 (****); ns (not significant). All data were shown as mean ± SD.

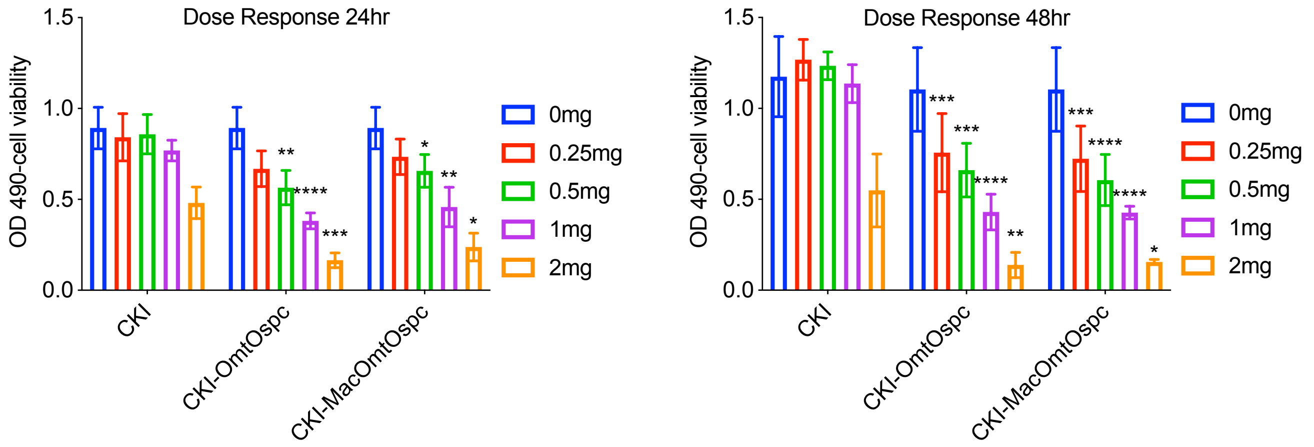

Supplementary Fig. 3: XTT cell viability assays of subtractive fractions CKI-OmtOspc and CKI-MacOmtOspc in MDA-MB-231 cells at 24- and 48-hour timepoints treated with four different concentrations ranging from 0.25 mg/ml to 2 mg/ml of CKI and equivalent concentrations of two other agents and 0 mg represents UT. Statistical tests were performed between drug treatments and UT (0mg). Statistically significant results shown as P < 0.05 (*) or P < 0.01 (**) P < 0.001 (***), or P < 0.0001 (****); ns (not significant). All data were shown as mean ± SD.

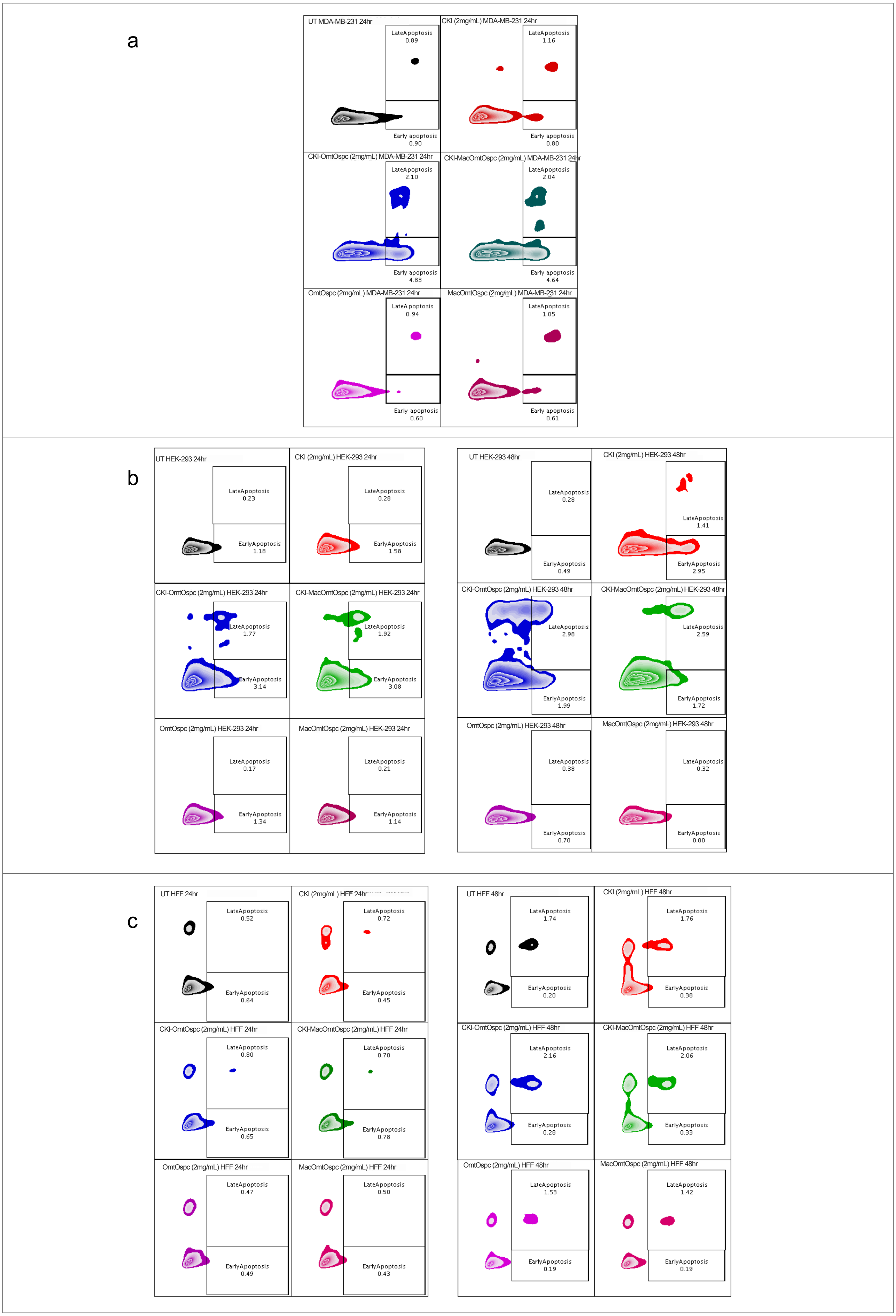

Supplementary Fig. 4: Representative plots of Annexin V and PI staining in (a) MDA-MB-231, (b) HEK-293, and (c) HFF.

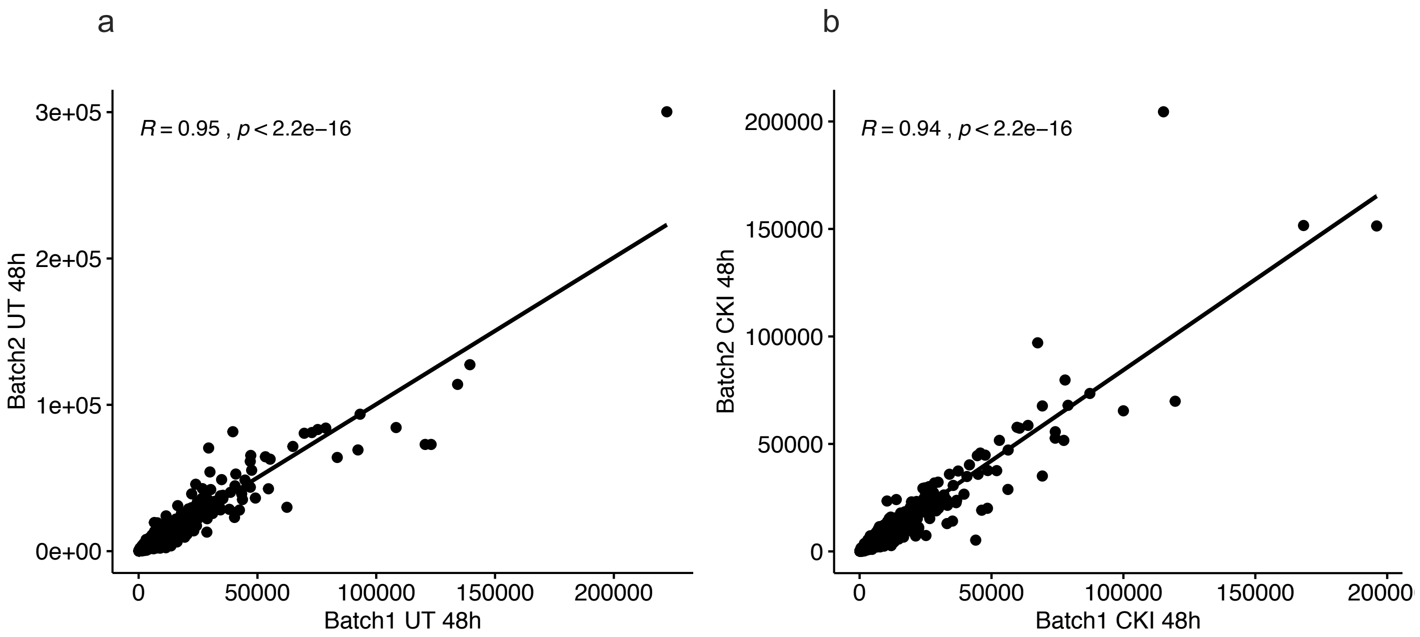

Supplementary Fig. 5: Correlation coefficient of (a) UT and (b) CKI of two batches at 48-hour treatments.

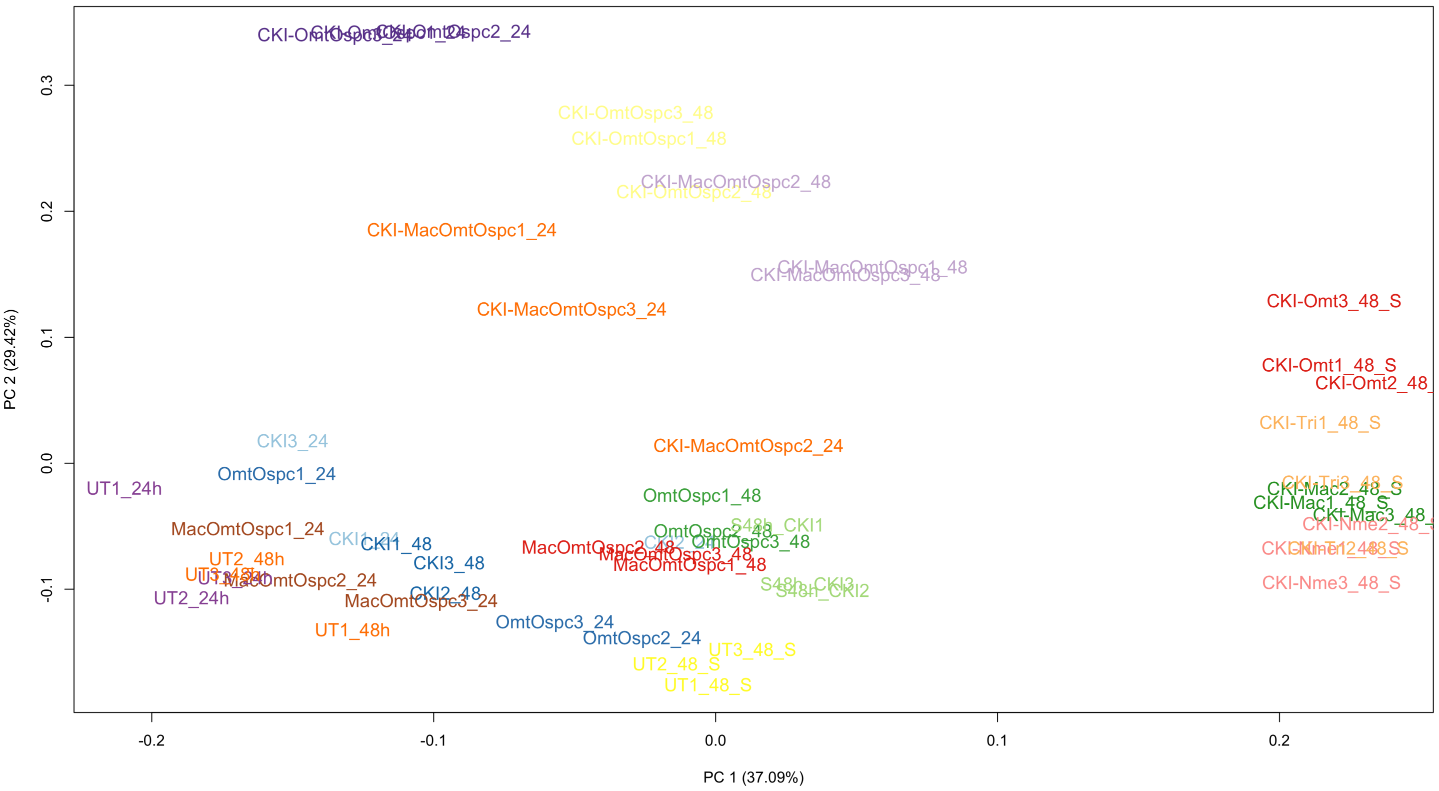

Supplementary Fig. 6: Multiple dimensional scaling (MDS) plot for samples based on expression profiles of all genes before the removal of unwanted variance (RUVs) in R package.

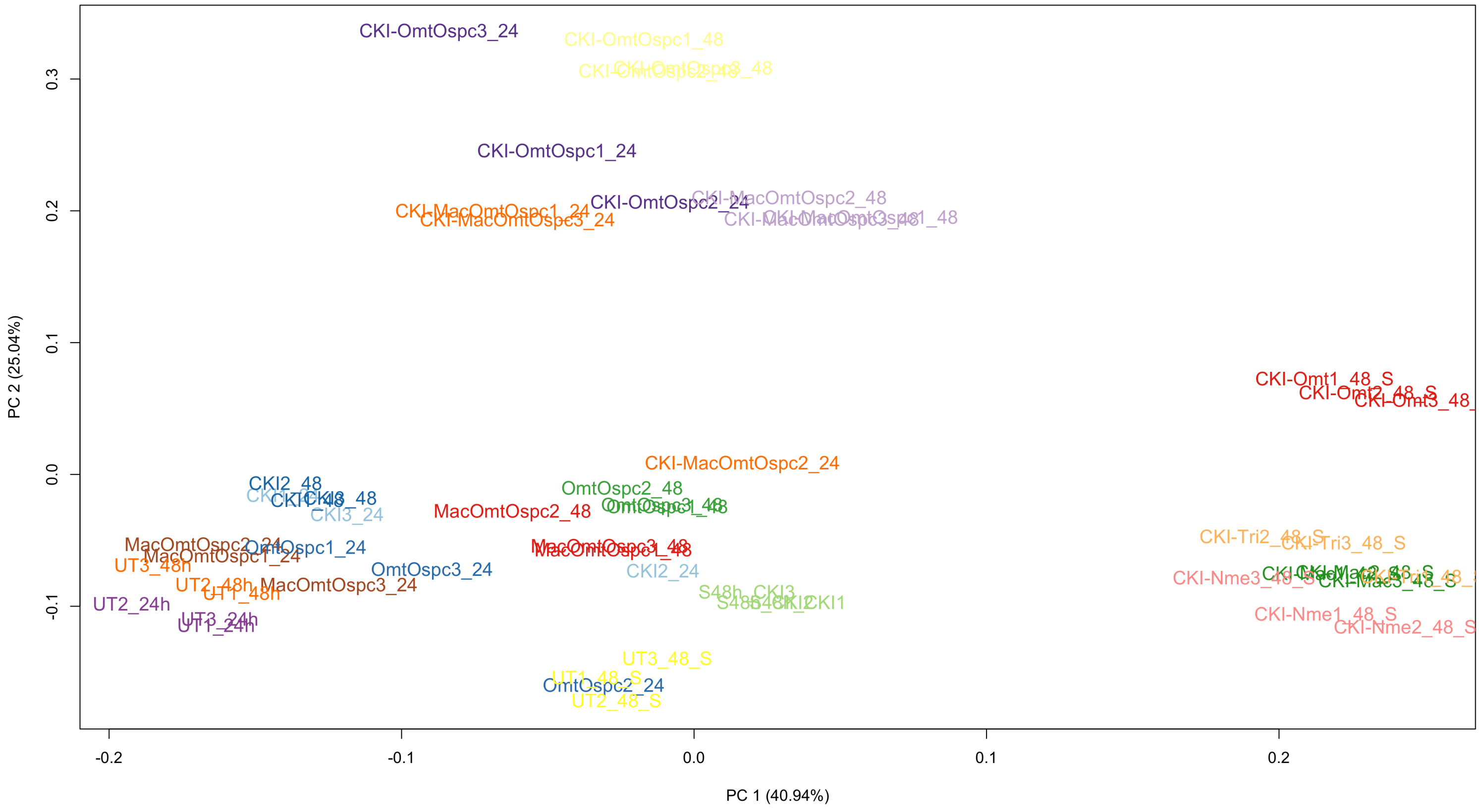

Supplementary Fig. 7: MDS plot for samples based on expression profiles of all genes after the application of RUVs.

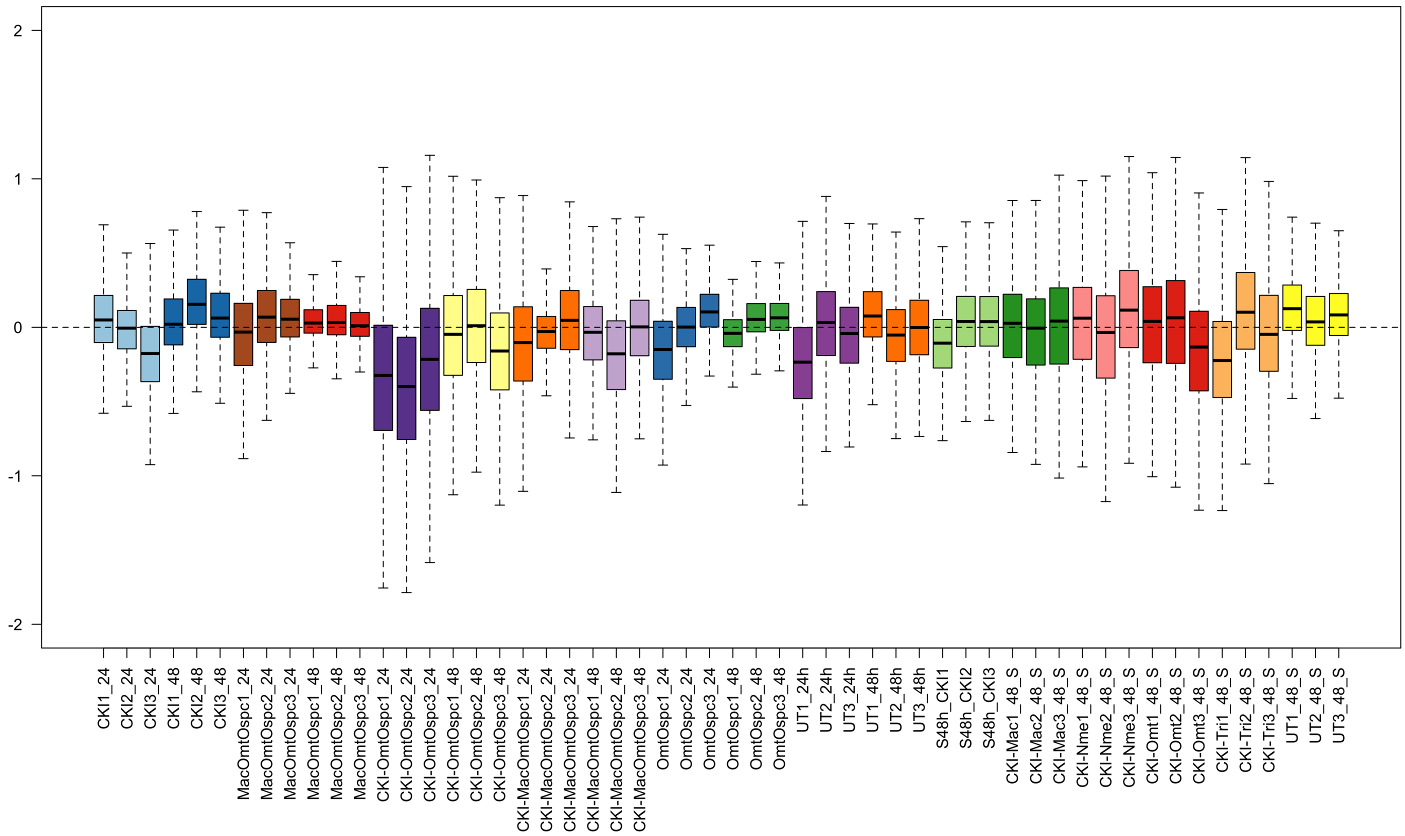

Supplementary Fig. 8: Box plot for samples based on expression profiles of all genes before the application of RUVs.

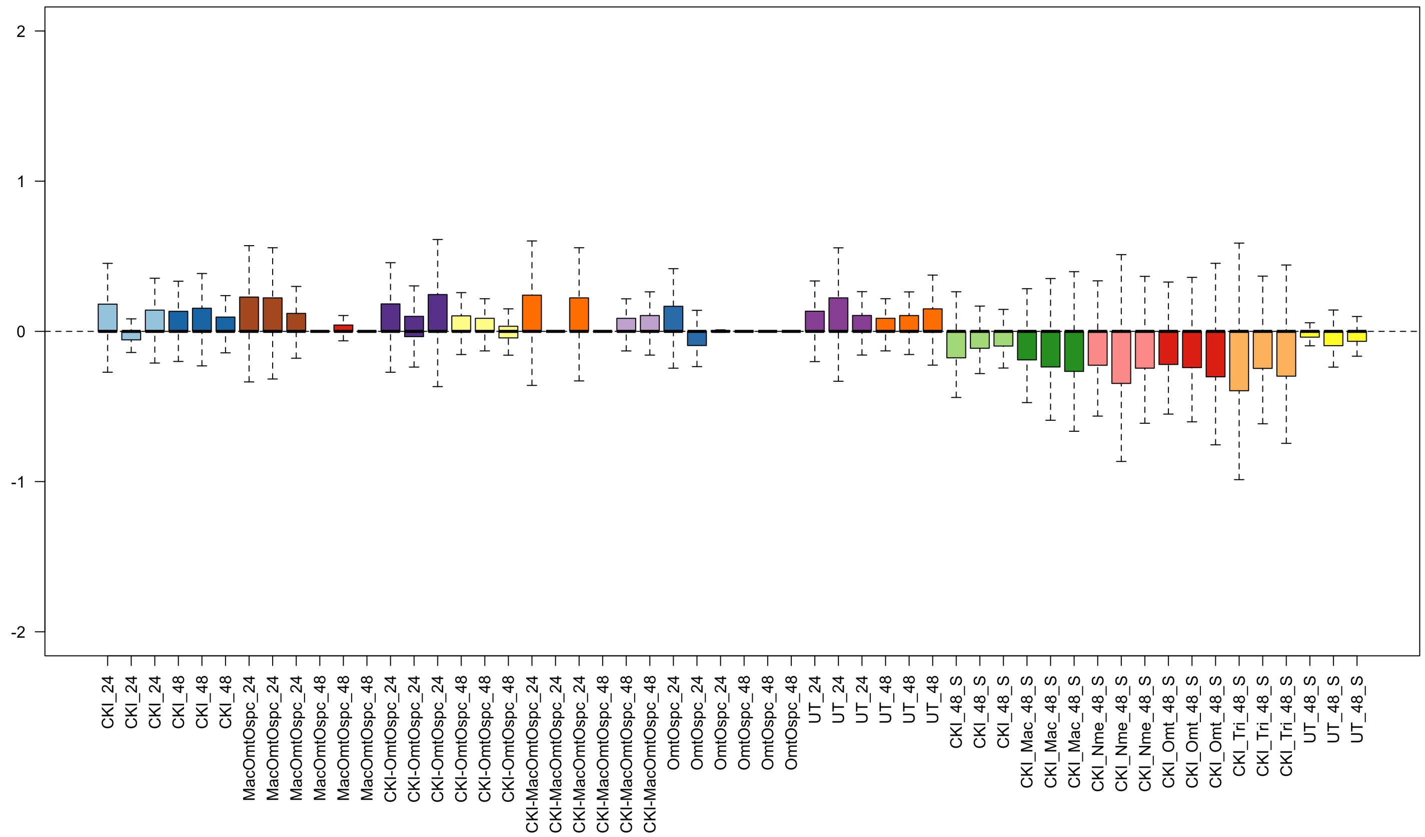

Supplementary Fig. 9: Box plot for samples based on expression profiles of all genes after the application of RUVs.

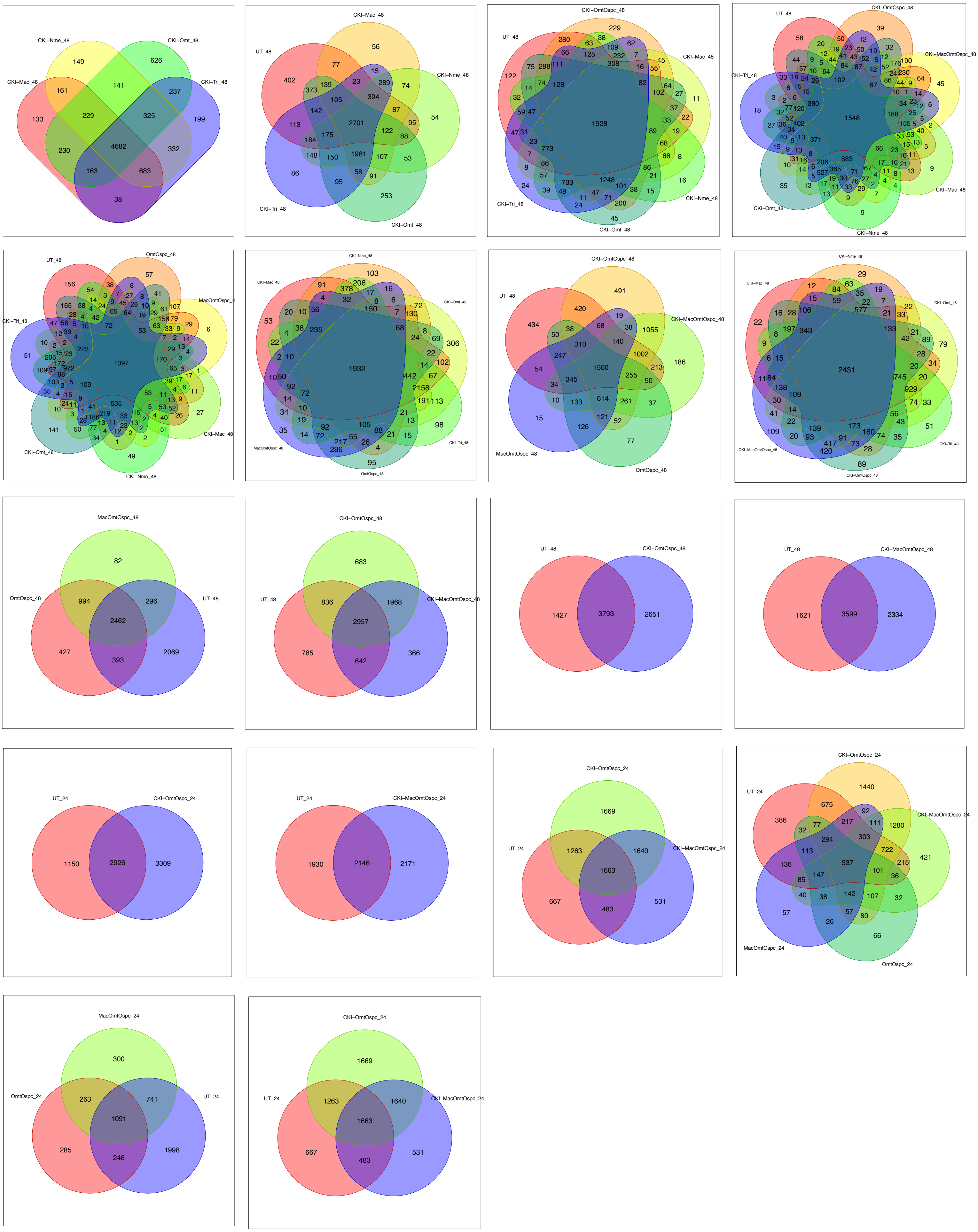

Supplementary Fig. 10: Venn diagrams showing the number of overlapping DE genes between treatments at 24-hour and 48-hour.

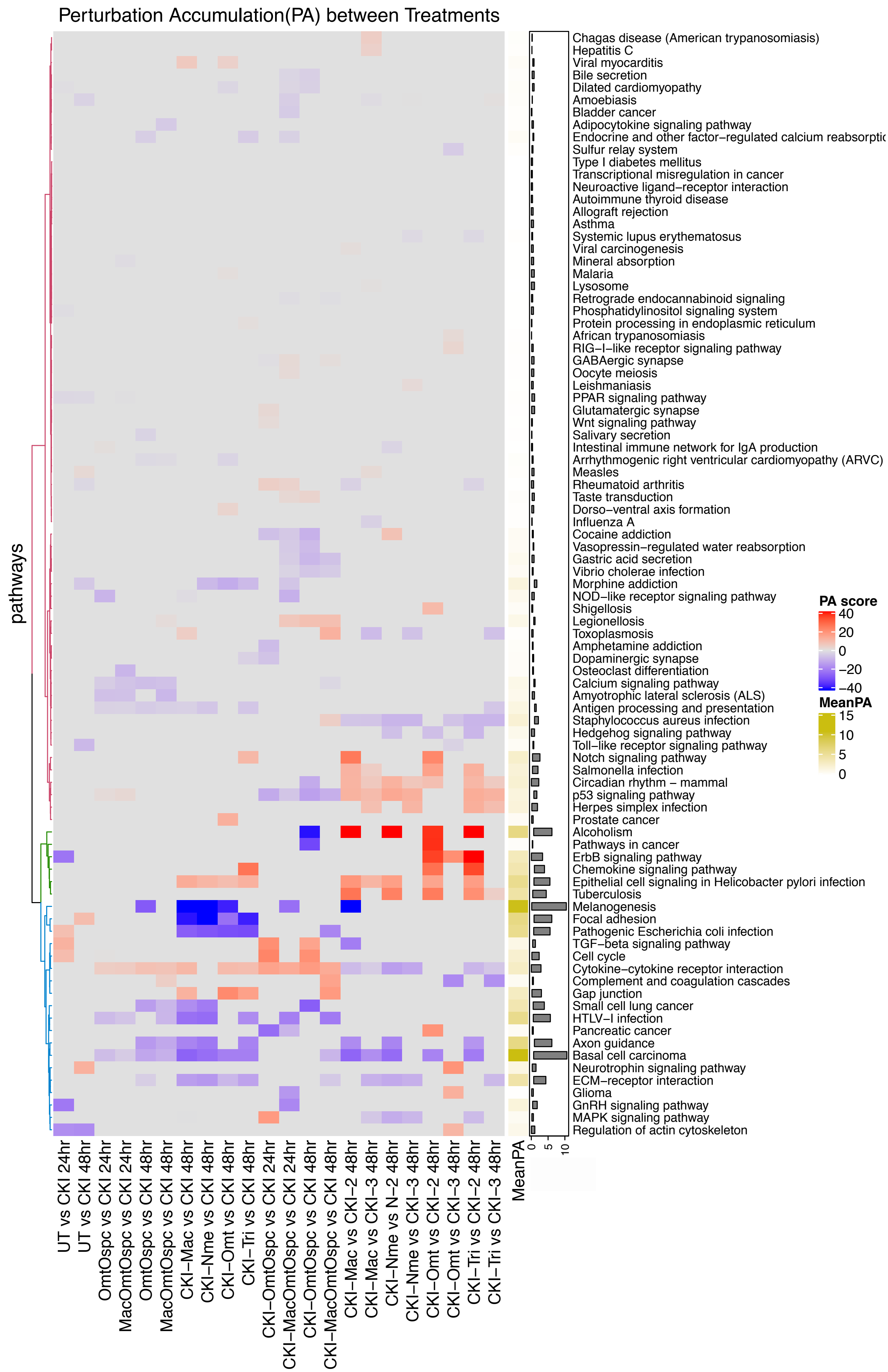

Supplementary Fig. 11: Identification of significantly perturbed pathways using SPIA (pG < 0.05) analysis. Eighty-nine significantly perturbed pathways from twenty-two comparisons were found.

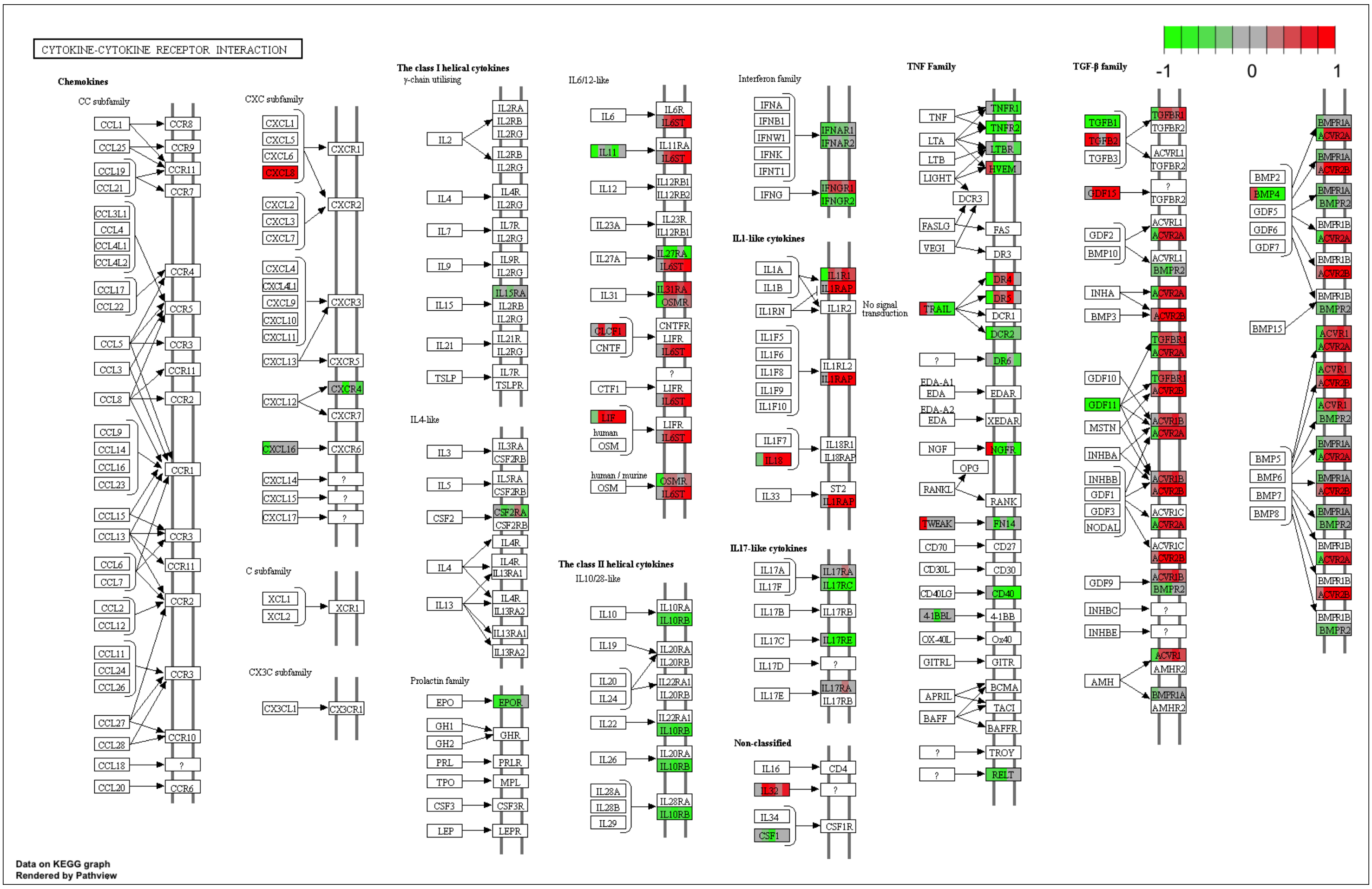

Supplementary Fig. 12: DE genes from the following comparisons (UT vs CKI, CKI-Mac vs CKI, CKI-Nme vs CKI, CKI-Omt vs CKI and CKI-Tri vs CKI) shown in "Cytokine-Cytokine Receptor Interaction pathway” at 48-hour. Significantly up- and down-regulated DE genes were coloured red and green respectively. Each coloured box was separated into five parts according to treatments in this order (from left to right): UT vs CKI, CKI-Mac vs CKI, CKI-Nme vs CKI, CKI-Omt vs CKI and CKI-Tri vs CKI. White or grey colours represented gene(s) that were not significantly differentially expressed by the treatments.

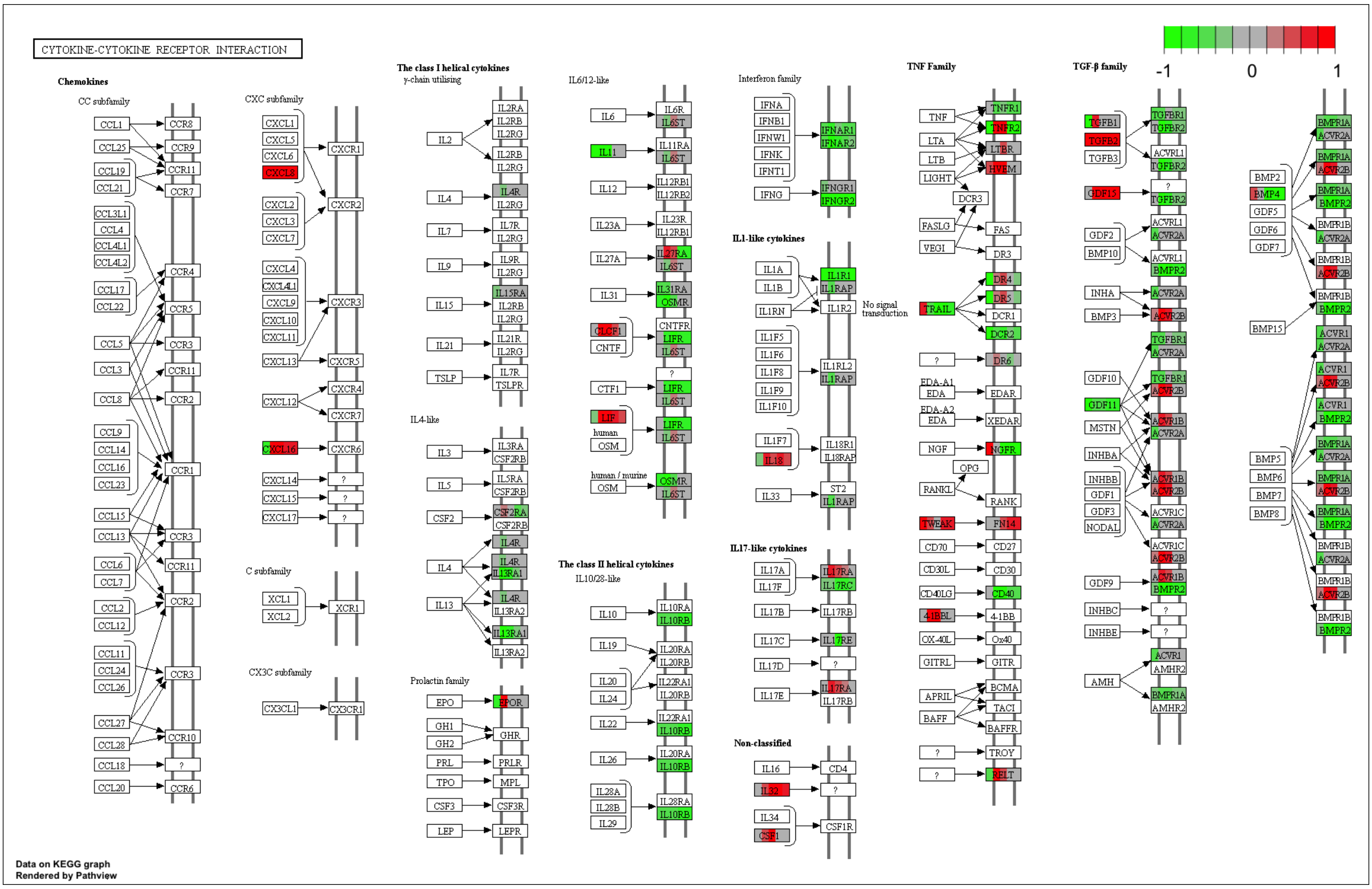

Supplementary Fig. 13: DE genes from the following comparisons (UT vs CKI, CKI-OmtOspc vs CKI, CKI-MacOmtOspc vs CKI, OmtOspc vs CKI and MacOmtOspc vs CKI) shown in the "Cytokine-Cytokine Receptor Interaction pathway" at 48-hour. Significantly up- and down-regulated DE genes were coloured red and green respectively. Each coloured box was separated into five parts according to this order (from left to right): UT vs CKI, CKI-OmtOspc vs CKI, CKI-MacOmtOspc vs CKI, OmtOspc vs CKI and MacOmtOspc vs CKI. White or grey colours represented gene(s) that were not significantly differentially expressed by the treatments.

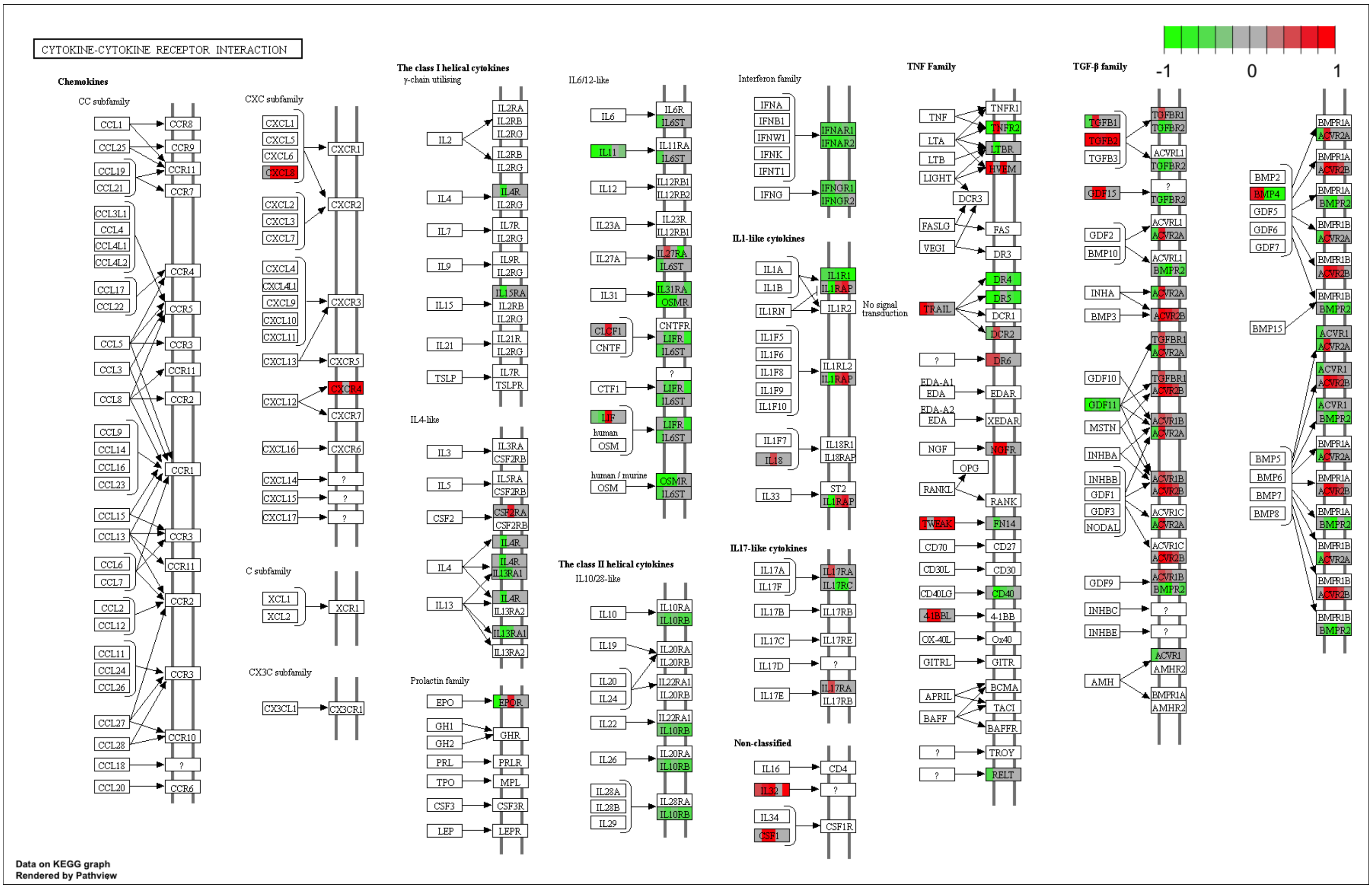

Supplementary Fig. 14: DE genes from the following comparisons (UT vs CKI, CKI-OmtOspc vs CKI, CKI-MacOmtOspc vs CKI, OmtOspc vs CKI and MacOmtOspc vs CKI) shown in the "Cytokine-Cytokine Receptor Interaction pathway" at 24-hour. Significantly up- and down-regulated DE genes were coloured red and green respectively. Each coloured box was separated into five parts according to this order (from left to right): UT vs CKI, CKI -OmtOspc vs CKI, CKI-MacOmtOspc vs CKI, OmtOspc vs CKI and MacOmtOspc vs CKI. White or grey colours represented gene(s) that were not significantly differentially expressed by the treatments.

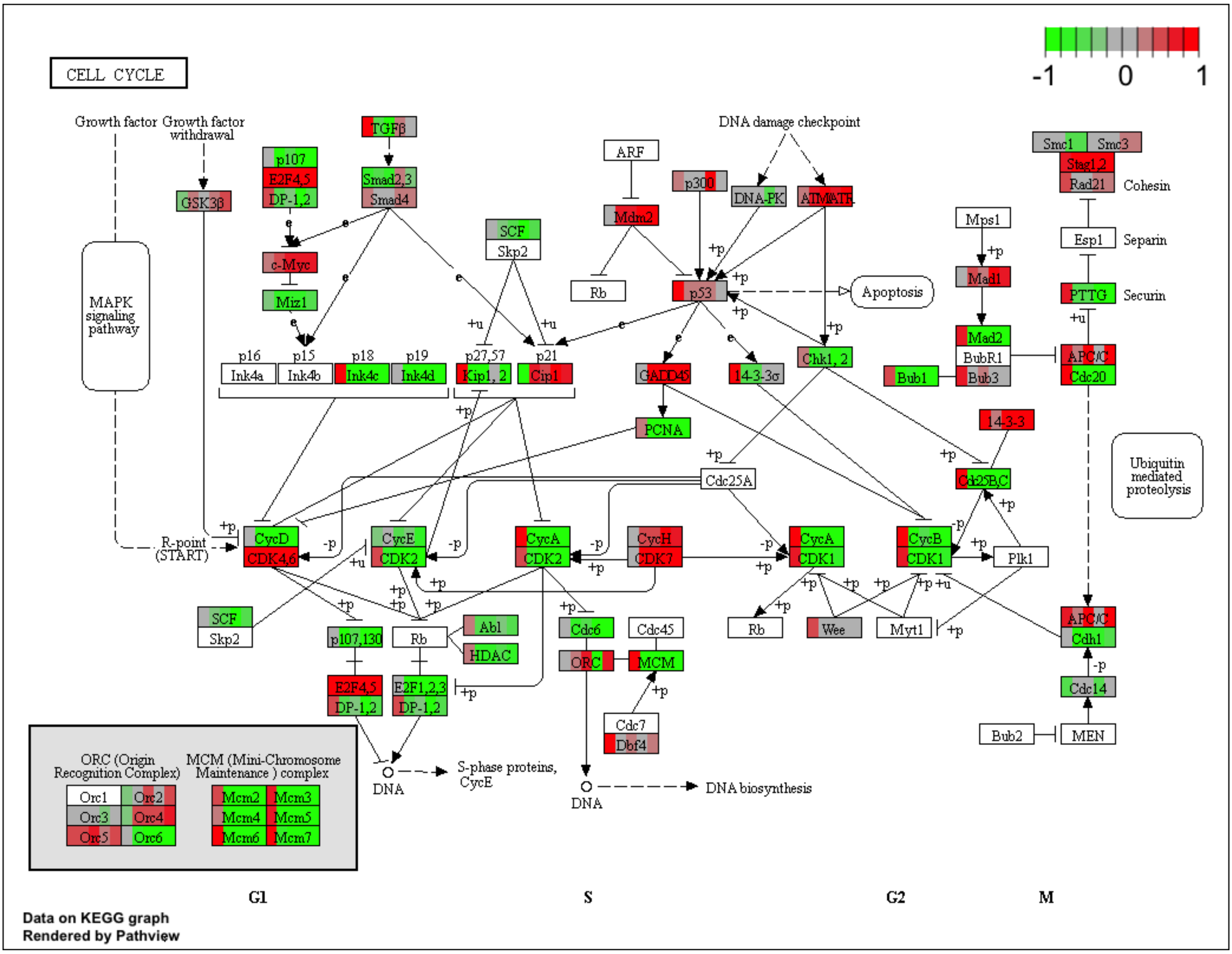

Supplementary Fig. 15: DE genes from the following comparisons (UT vs CKI, CKI -Mac vs CKI, CKI-Nme vs CKI, CKI-Omt vs CKI and CKI-Tri vs CKI) shown in the "Cell-Cycle pathway" at 48-hour. Significantly up- and down-regulated DE genes were coloured with red and green respectively. Each coloured box was separated into five parts according to this order (from left to right): UT vs CKI, CKI -Mac vs CKI, CKI-Nme vs CKI, CKI-Omt vs CKI and CKI-Tri vs CKI. White or grey colours represented gene(s) that were not significantly differentially expressed by the treatments.

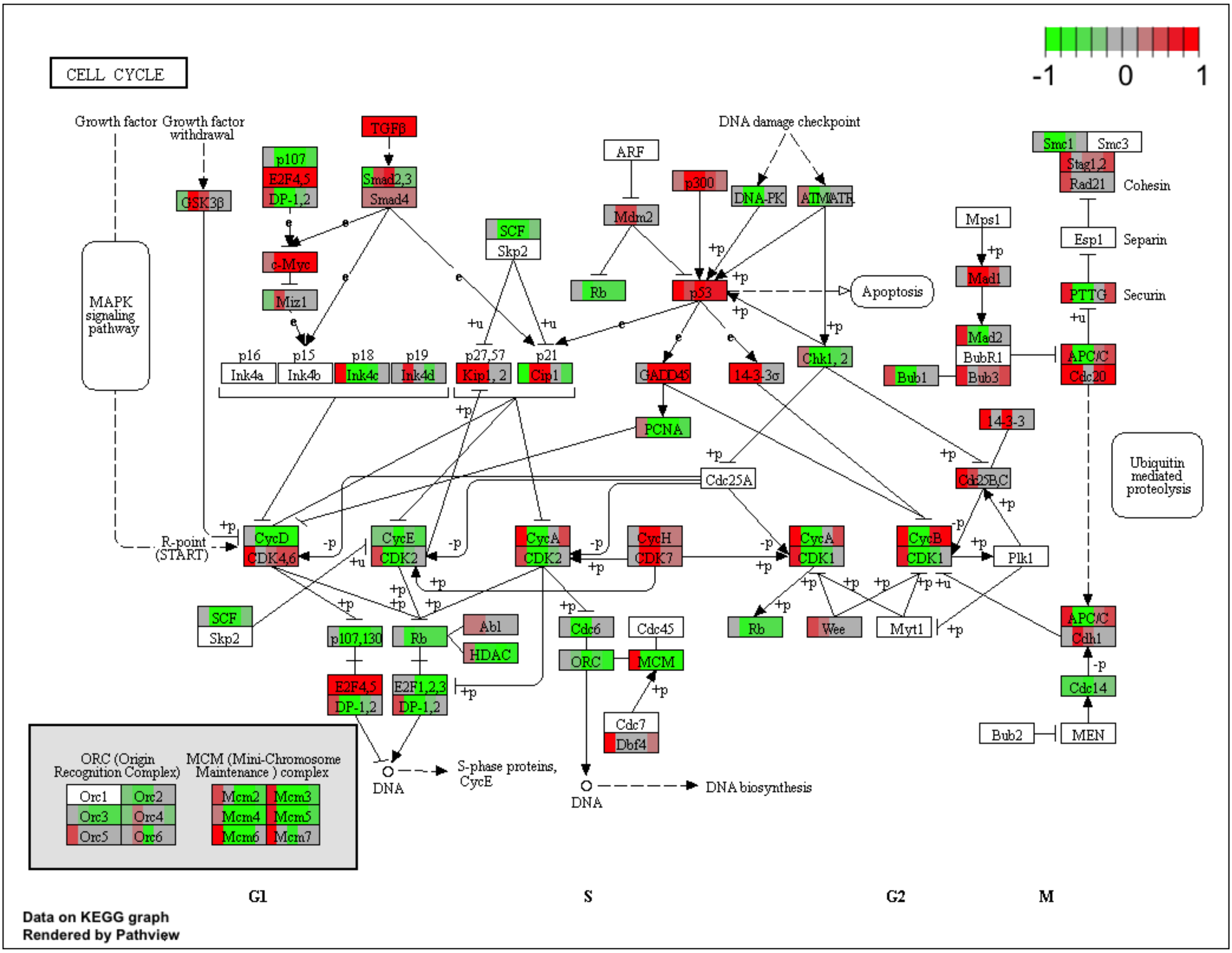

Supplementary Fig. 16: DE genes from the following comparisons (UT vs CKI, CKI-OmtOspc vs CKI, CKI-MacOmtOspc vs CKI, OmtOspc vs CKI and MacOmtOspc vs CKI) shown in the “Cell-Cycle pathway” at 48-hour treatments. Significantly up- and down-regulated DE genes were coloured red and green respectively. Each coloured box was separated into five parts according to this order (from left to right): UT vs CKI, CKI-OmtOspc vs CKI, CKI-MacOmtOspc vs CKI, OmtOspc vs CKI and MacOmtOspc vs CKI. White or grey colours represented gene(s) that were not significantly differentially expressed by the treatments.

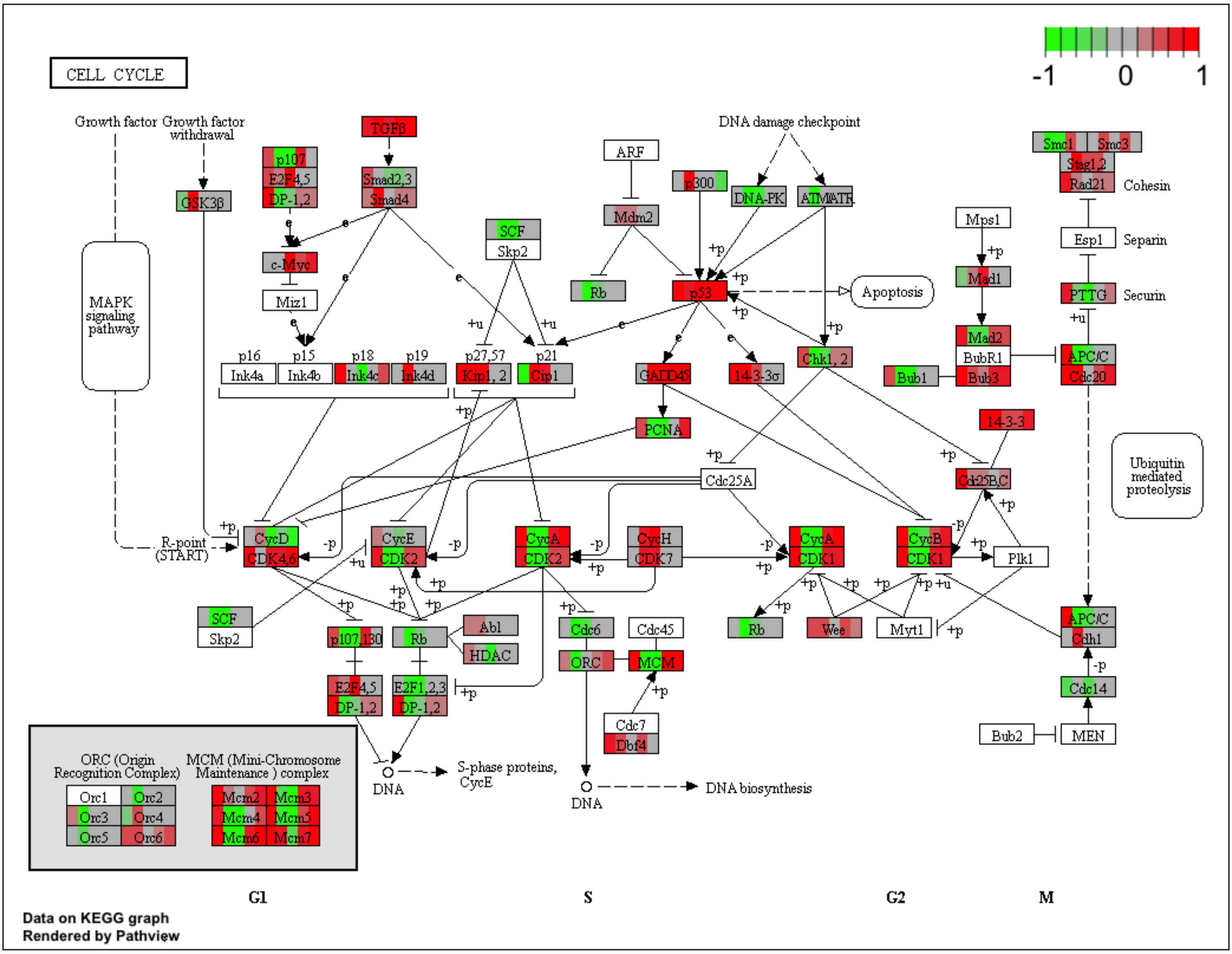

Supplementary Fig. 17: DE genes from the following comparisons (UT vs CKI, CKI-OmtOspc vs CKI, CKI-MacOmtOspc vs CKI, OmtOspc vs CKI and MacOmtOspc vs CKI) shown in the "Cell-Cycle pathway" at 24-hour. Significantly up- and down-regulated DE genes were coloured red and green respectively. Each coloured box was separated into five parts according to this order (from left to right): UT vs CKI, CKI-OmtOspc vs CKI, CKI-MacOmtOspc vs CKI, OmtOspc vs CKI and MacOmtOspc vs CKI. White or grey colours represented gene(s) that were not significantly differentially expressed by the treatments.

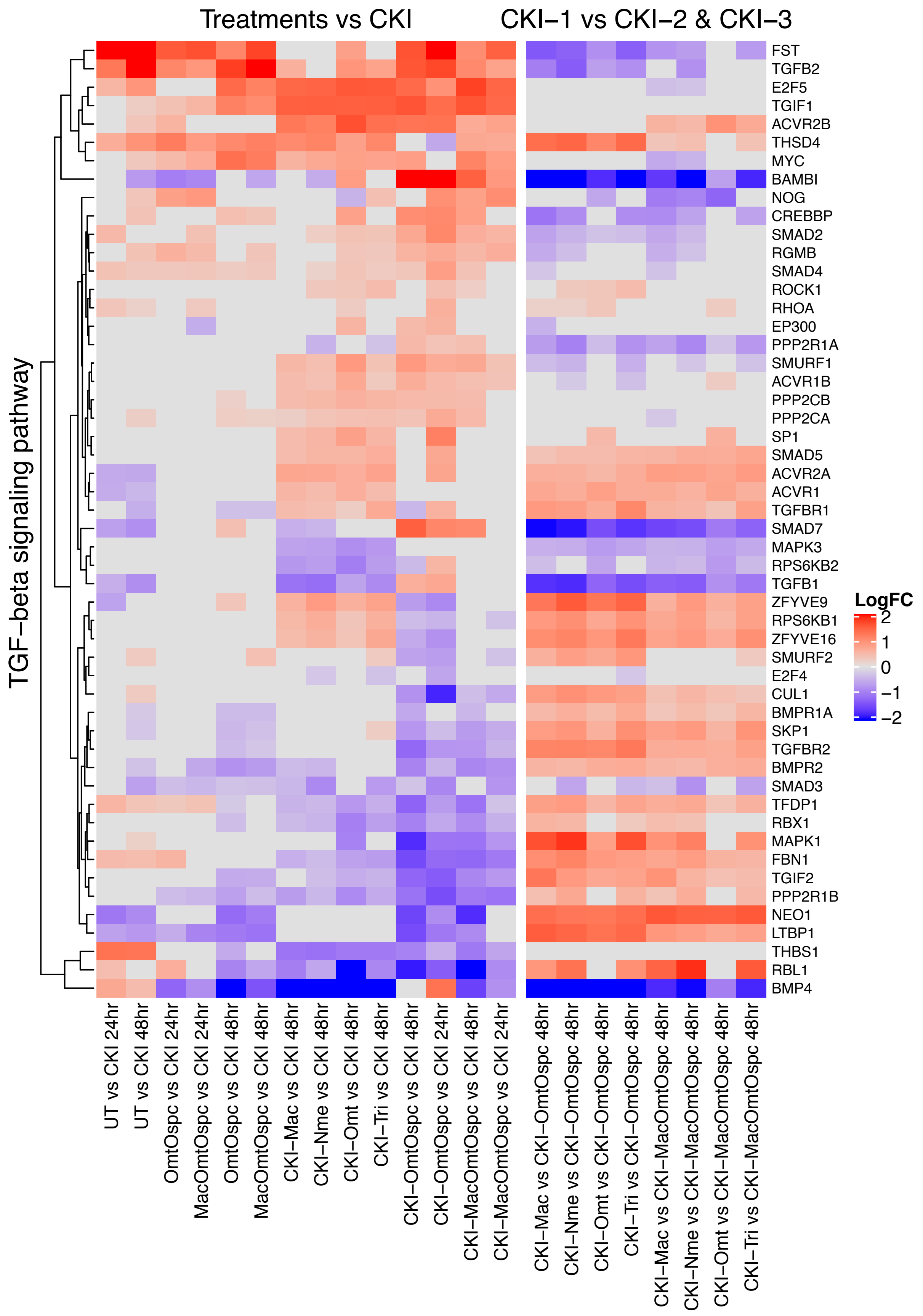

Supplementary Fig. 18: Differential gene expression profiles of all treatments for TGF-ß signalling pathway: the left panel shows comparison of subtractive fraction treated cells against CKI treatment and the right panel shows comparison of single compound subtractive fraction treated cells against the treatments for two and three compound subtractive fractions.

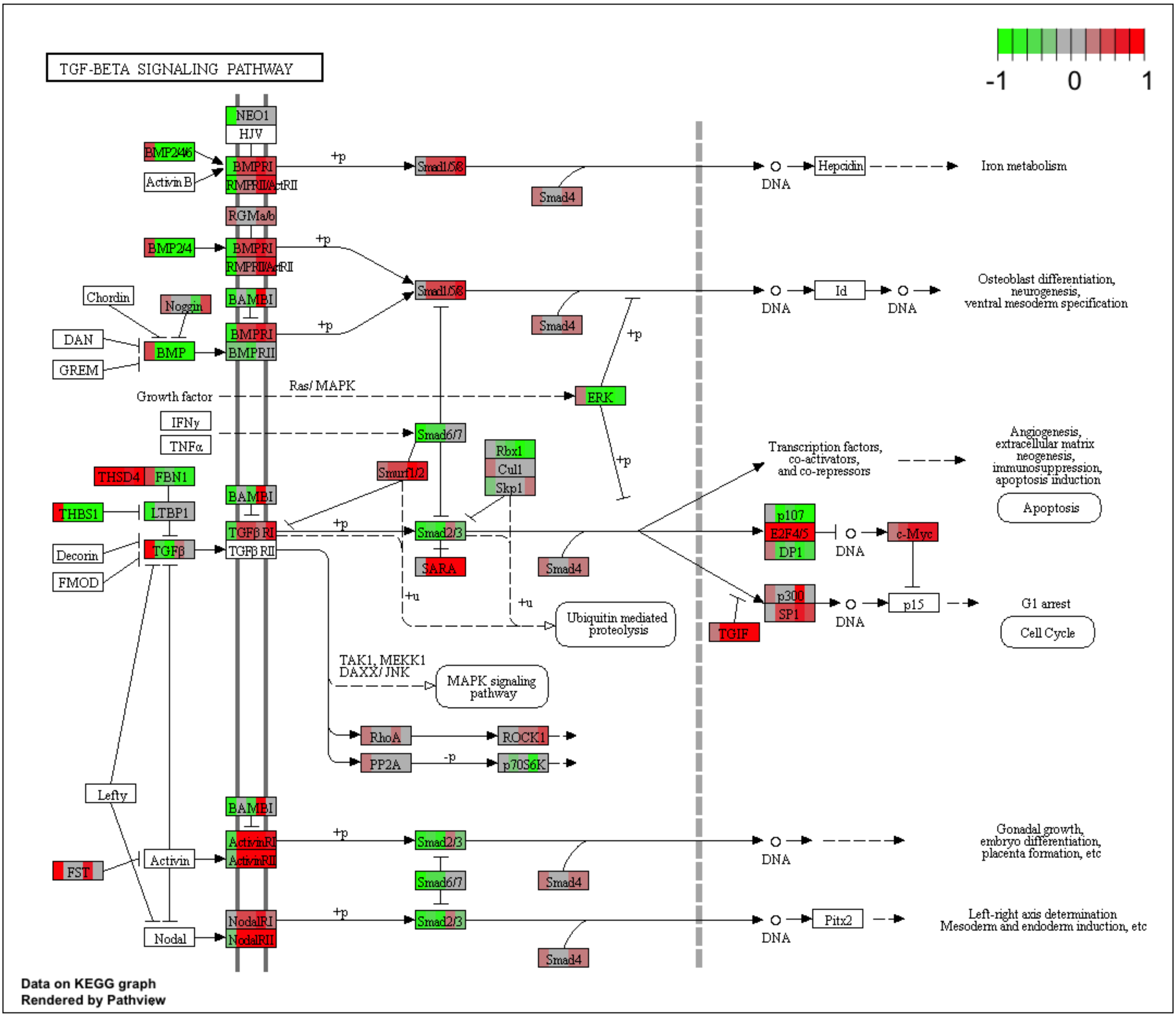

Supplementary Fig. 19: DE genes from the following comparisons (UT vs CKI, CKI-Mac vs CKI, CKI-Nme vs CKI, CKI-Omt vs CKI and CKI-Tri vs CKI) shown in the "TGF-ß signalling pathway" at 48-hour. Significantly up- and down-regulated DE genes were coloured red and green respectively. Each coloured box was separated into five parts according to this order (from left to right): UT vs CKI, CKI-Mac vs CKI, CKI-Nme vs CKI, CKI-Omt vs CKI and CKI-Tri vs CKI. White or grey colours represented gene(s) that were not significantly differentially expressed by the treatments.

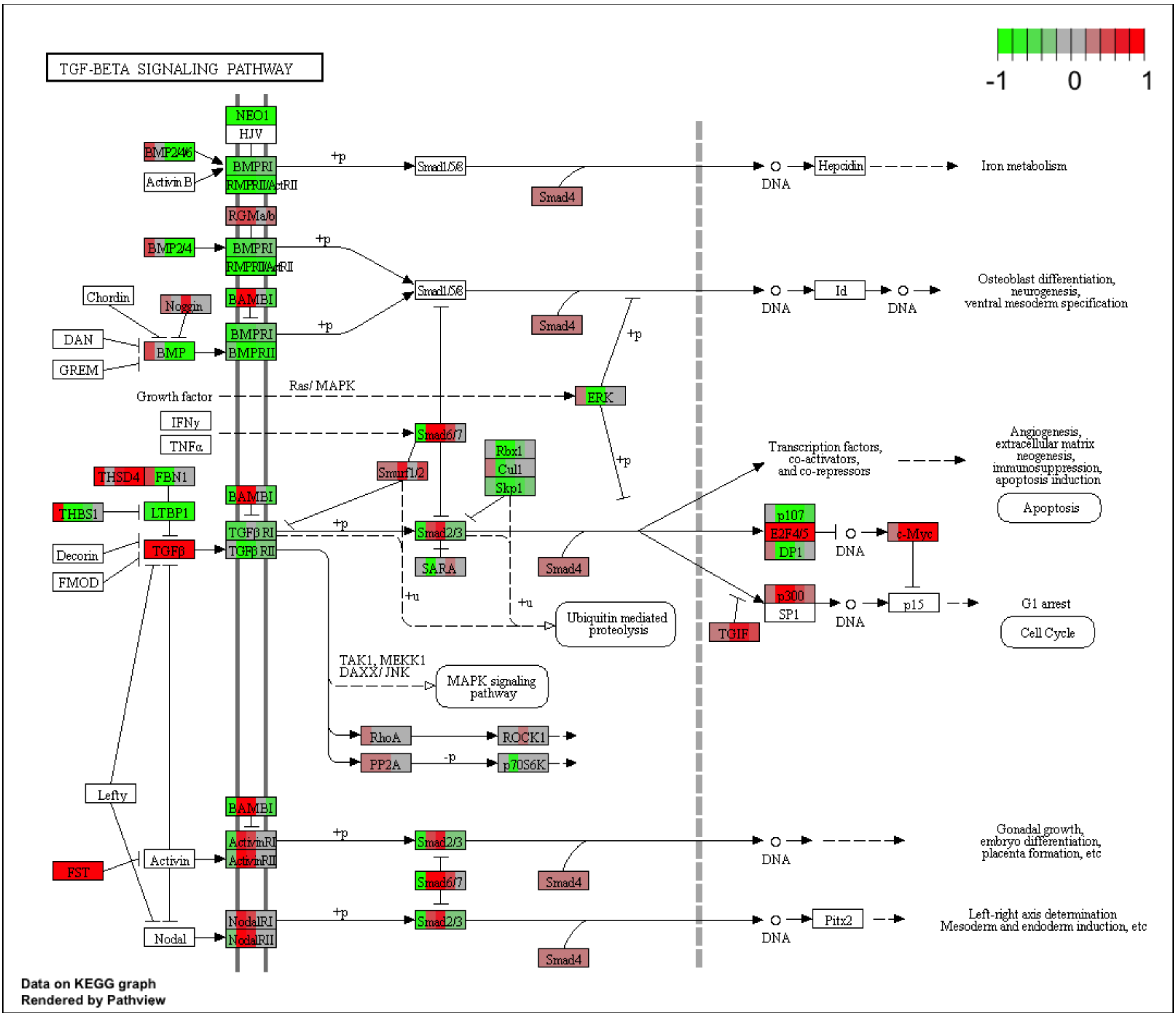

Supplementary Fig. 20: DE genes from the following comparisons (UT vs CKI, CKI-OmtOspc vs CKI, CKI-MacOmtOspc vs CKI, OmtOspc vs CKI and MacOmtOspc vs CKI) shown in the “TGF-ß signalling pathway” at 48-hour treatments. Significantly up- and down-regulated DE genes were coloured red and green respectively. Each coloured box was separated into five parts according to this order (from left to right): UT vs CKI, CKI-OmtOspc vs CKI, CKI-MacOmtOspc vs CKI, OmtOspc vs CKI and MacOmtOspc vs CKI. White or grey colours represented gene(s) that were not significantly differentially expressed by the treatments.

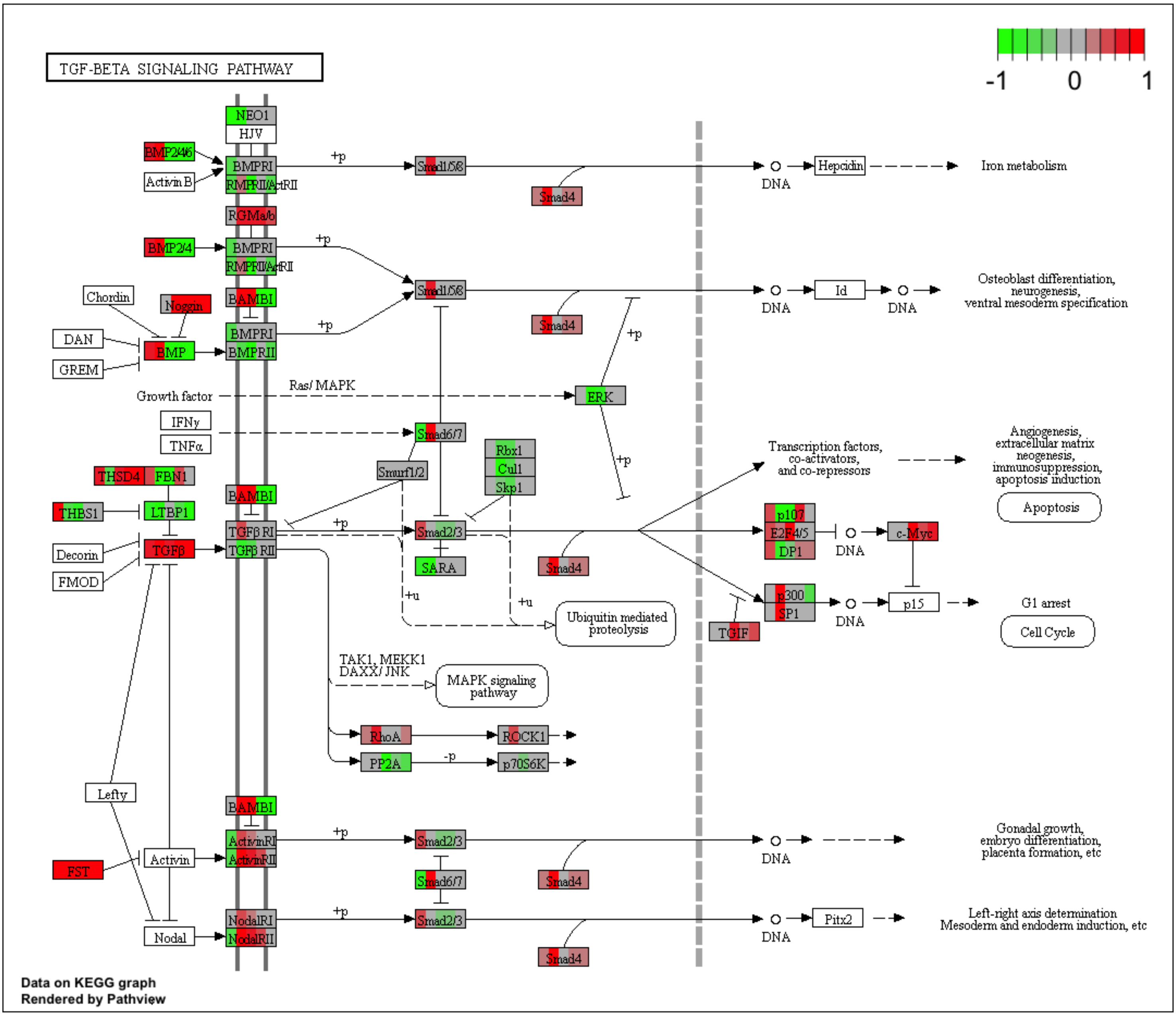

Supplementary Fig. 21: DE genes from the following comparisons (UT vs CKI, CKI-OmtOspc vs CKI, CKI-MacOmtOspc vs CKI, OmtOspc vs CKI and MacOmtOspc vs CKI) shown in the "TGF-ß signalling pathway" at 24-hour. Significantly up- and down-regulated DE genes were coloured red and green respectively. Each coloured box was separated into five parts according to this order (from left to right): UT vs CKI, CKI-OmtOspc vs CKI, CKI-MacOmtOspc vs CKI, OmtOspc vs CKI and MacOmtOspc vs CKI. White or grey colours represented gene(s) that were not significantly differentially expressed by the treatments.

Supplementary Table 1: Summary of the samples, number of samples, RNA-Seq sample names, processed sample names and treatments

Number of Samples	RNA-Seq Sample Names	Processed Sample Names	Treatments
Sample 1 (Batch1)	24h_UT1_Rep1_Batch1	UT1 24	Untreated
Sample 2 (Batch1)	24h_UT2_Rep2_Batch1	UT2 24	Untreated
Sample 3 (Batch1)	24h_UT3_Rep3_Batch1	UT3 24	Untreated
Sample 4 (Batch1)	24h_2mg_CKI_Rep1_Batch1	CKI1 24	2mg (1/13.25 dilution) CKI 24hr
Sample 5 (Batch1)	24h_2mg_CKI1_Rep2_Batch1	CKI2 24	2mg (1/13.25 dilution) CKI 24hr
Sample 6 (Batch1)	24h_2mg_CKI3_Rep3_Batch1	CKI3 24	2mg (1/13.25 dilution) CKI 24hr
Sample 7 (Batch1)	24h_2mg_N_2_1_Rep1_Batch1	CKI-OmtOspc 1 24	1/13.25 dilution CKI-OmtOspc 24hr
Sample 8 (Batch1)	24h_2mg_N_2_2_Rep2_Batch1	CKI-OmtOspc 2 24	1/13.25 dilution CKI-OmtOspc 24hr
Sample 9 (Batch1)	24h_2mg_N_2_3_Rep3_Batch1	CKI-OmtOspc 3 24	1/13.25 dilution CKI-OmtOspc 24hr
Sample 10 (Batch1)	24h_2mg_N_3_1_Rep1_Batch1	CKI-MacOmtOspc 1 24	1/13.25 dilution CKI-MacOmtOspc 24hr
Sample 11 (Batch1)	24h_2mg_N_3_Rep2_Batch1	CKI-MacOmtOspc 2 24	1/13.25 dilution CKI-MacOmtOspc 24hr
Sample 12 (Batch1)	24h_2mg_N_3_3_Rep3_Batch1	CKI-MacOmtOspc 3 24	11/13.25 dilution CKI-MacOmtOspc 24hr
Sample 13 (Batch1)	24h_2mg_OO_1_Rep1_Batch1	OmtOspc 1 24	1/13.25 dilution OmtOspc 24hr
Sample 14 (Batch1)	24h_2mg_OO_Rep2_Batch1	OmtOspc 2 24	1/13.25 dilution OmtOspc 24hr
Sample 15 (Batch1)	24h_2mg_OO_3_Rep3_Batch1	OmtOspc 3 24	1/13.25 dilution OmtOspc 24hr
Sample 16 (Batch1)	24h_2mg_MOO_1_Rep1_Batch1	MacOmtOspc 1 24	1/13.25 dilution MacOmtOspc 24hr
Sample 17 (Batch1)	24h_2mg_MOO_2_Rep2_Batch1	MacOmtOspc 2 24	1/13.25 dilution MacOmtOspc 24hr
Sample 18 (Batch1)	24h_2mg_MOO_3_Rep3_Batch1	MacOmtOspc 3 24	1/13.25 dilution MacOmtOspc 24hr
Sample 19 (Batch1)	48h_UT1_Rep1_Batch1	UT1 48	Untreated
Sample 20 (Batch1)	48h_UT2_Rep2_Batch1	UT2 48	Untreated
Sample 21 (Batch1)	48h_UT3_Rep3_Batch1	UT3 48	Untreated
Sample 22 (Batch1)	48h_2mg_CKI1_Rep1_Batch1	CKI1 48	2mg (1/13.25 dilution) CKI 48hr
Sample 23 (Batch1)	48h_2mg_CKI2_Rep2_Batch1	CKI2 48	2mg (1/13.25 dilution) CKI 48hr
Sample 24 (Batch1)	48h_2mg_CKI3_Rep3_Batch1	CKI3 48	2mg (1/13.25 dilution) CKI 48hr
Sample 25(Batch1)	48h_2mg_N_2_1_Rep1_Batch1	CKI-OmtOspc 1 48	1/13.25 dilution CKI-OmtOspc 48hr
Sample 26 (Batch1)	48h_2mg_N_2_2_Rep2_Batch1	CKI-OmtOspc 2 48	1/13.25 dilution CKI-OmtOspc 48hr
Sample 27 (Batch1)	48h_2mg_N_2_3_Rep3_Batch1	CKI-OmtOspc 3 48	1/13.25 dilution CKI-OmtOspc 48hr
Sample 28 (Batch1)	48h_2mg_N_3_1_Rep1_Batch1	CKI-MacOmtOspc 1 48	1/13.25 dilution CKI-MacOmtOspc 48hr
Sample 29 (Batch1)	48h_2mg_N_3_2_Rep2_Batch1	CKI-MacOmtOspc 2 48	1/13.25 dilution CKI-MacOmtOspc 48hr

Sample 30 (Batch1)	48h_2mg_N_3_3_Rep3_Batch1	CKI-MacOmtOspc 3 48	1/13.25 dilution CKI-MacOmtOspc 48hr
Sample 31 (Batch1)	48h_2mg_OO_1_Rep1_Batch1	OmtOspc 1 48	1/13.25 dilution OmtOspc 48hr
Sample 32 (Batch1)	48h_2mg_OO_2_Rep2_Batch1	OmtOspc 2 48	1/13.25 dilution OmtOspc 48hr
Sample 33 (Batch1)	48h_2mg_OO_3_Rep3_Batch1	OmtOspc 3 48	1/13.25 dilution OmtOspc 48hr
Sample 34 (Batch1)	48h_2mg_MOO_1_Rep1_Batch1	MacOmtOspc 1 48	1/13.25 dilution MacOmtOspc 48hr
Sample 35 (Batch1)	48h_2mg_MOO_2_Rep2_Batch1	MacOmtOspc 2 48	1/13.25 dilution MacOmtOspc 48hr
Sample 36 (Batch1)	48h_2mg_MOO_3_Rep3_Batch1	MacOmtOspc 3 48	1/13.25 dilution MacOmtOspc 48hr
Sample 37 (Batch2)	48h_UT1_Rep1_Batch2	UT1 48 S	Untreated
Sample 38 (Batch2)	48h_UT2_Rep2_Batch2	UT2 48 S	Untreated
Sample 39 (Batch2)	48h_UT3_Rep3_Batch2	UT3 48 S	Untreated
Sample 40 (Batch2)	48h_2mg_CKI1_Rep1_Batch2	CKI1 48 S	2mg (1/13.25 dilution) CKI 48hr
Sample 41 (Batch2)	48h_2mg_CKI2_Rep2_Batch2	CKI2 48 S	2mg (1/13.25 dilution) CKI 48hr
Sample 42 (Batch2)	48h_2mg_CKI3_Rep3_Batch2	CKI3 48 S	2mg (1/13.25 dilution) CKI 48hr
Sample 43 (Batch2)	48h_2mg_N_Mac1_Rep1_Batch2	CKI-Mac 1 48 S	1/13.25 dilution CKI-Mac 48hr
Sample 44 (Batch2)	48h_2mg_N_Mac2_Rep2_Batch2	CKI-Mac 2 48 S	1/13.25 dilution CKI-Mac 48hr
Sample 45 (Batch2)	48h_2mg_N_Mac3_Rep3_Batch2	CKI-Mac 3 48 S	1/13.25 dilution CKI-Mac 48hr
Sample 46 (Batch2)	48h_2mg_N_Nme1_Rep1_Batch2	CKI-Nme 1 48 S	1/13.25 dilution CKI-Nme 48hr
Sample 47 (Batch2)	48h_2mg_N_Nme2_Rep2_Batch2	CKI-Nme 2 48 S	1/13.25 dilution CKI-Nme 48hr
Sample 48 (Batch2)	48h_2mg_N_Nme3_Rep3_Batch2	CKI-Nme 3 48 S	1/13.25 dilution CKI-Nme 48hr
Sample 49 (Batch2)	48h_2mg_N_Omt1_Rep1_Batch2	CKI-Omt 1 48 S	1/13.25 dilution CKI-Omt 48hr
Sample 50 (Batch2)	48h_2mg_N_Omt2_Rep2_Batch2	CKI-Omt 2 48 S	1/13.25 dilution CKI-Omt 48hr
Sample 51 (Batch2)	48h_2mg_N_Omt3_Rep3_Batch2	CKI-Omt 3 48 S	1/13.25 dilution CKI-Omt 48hr
Sample 52 (Batch2)	48h_2mg_N_Tri1_Rep1_Batch2	CKI-Tri 1 48 S	1/13.25 dilution CKI-Tri 48hr
Sample 53 (Batch2)	48h_2mg_N_Tri2_Rep2_Batch2	CKI-Tri 2 48 S	1/13.25 dilution CKI-Tri 48hr
Sample 54 (Batch2)	48h_2mg_N_Tri3_Rep3_Batch2	CKI-Tri 3 48 S	1/13.25 dilution CKI-Tri 48hr

Supplementary Table 2: Summary of shared, differentially expressed (DE) genes across treatments. Similarity (%) calculated from total number of shared DE genes from all listed comparisons. To find the number of DE genes, CKI treatment was used as a baseline to compare all other fractionated treatments in order to emphasize the effect of depleted compounds and UT (untreated) was used as a base to calculate the DE genes for CKI treatment.

Treatments Comparisons	Similarity (%)	Time course (hours)
4 x (CKI-1)	71.3	48
4 x (CKI -1) and CKI	42.7	48
4 x (CKI -1), CKI and CKI -OmtOspc	30.4	48
4 x (CKI -1), CKI, CKI -OmtOspc and CKI -MacOmtOspc	24.6	48
4 x (CKI -1), CKI, OmtOspc, and MacOmtOspc	24.6	48
4 x (CKI -1), OmtOspc, and MacOmtOspc	33.7	48
4 x (CKI -1), CKI -OmtOspc and CKI -MacOmtOspc	37.7	48
CKI and CKI -OmtOspc	64.4	48
CKI and CKI -MacOmtOspc	63.9	48
CKI, CKI -OmtOspc and CKI -MacOmtOspc	50.1	48
CKI, CKI -OmtOspc, CKI -MacOmtOspc, OmtOspc, and MacOmtOspc	30.2	48
UT, OmtOspc, and MacOmtOspc	54.9	48
CKI and CKI -OmtOspc	56.0	24
CKI and CKI -MacOmtOspc	45.6	24
CKI, CKI -OmtOspc and CKI -MacOmtOspc	31.6	24
CKI, CKI -OmtOspc, CKI -MacOmtOspc, OmtOspc, and MacOmtOspc	13.3	24
UT, OmtOspc, and MacOmtOspc	39.1	24
